# UPLC-MS/MS Based Identification of Dietary Steryl Glucosides by Investigation of Corresponding Free Sterols

**DOI:** 10.3389/fchem.2018.00342

**Published:** 2018-08-22

**Authors:** Linda H. Münger, Samy Boulos, Laura Nyström

**Affiliations:** Laboratory of Food Biochemistry, Institute of Food, Nutrition and Health, Department of Health Sciences and Technology, ETH Zürich, Zurich, Switzerland

**Keywords:** steryl glucosides, free sterols, MS/MS, electrospray ionization, liquid chromatography, monomethyl steryl glucoside

## Abstract

Dietary plant foods are characterized by a vast molecular diversity of glycosylated sterols (SG) that differ in the structure of the steryl backbone. The identification of these polar steryl conjugates represents a major challenge as they are structurally highly similar, and commercial standards are limited to a few naturally abundant species. Spectral databases do not yet contain MS/MS spectra of these sterol conjugates obtained by electrospray ionization (ESI), which would facilitate their reliable identification. Thus, this study aimed at providing novel information on ESI-MS/MS spectra of both abundant and minor SG found in foods. As a first step, however, free sterols (FS) were investigated for their fragmentation behavior as they share the same intermediate ion as SG. Pure SG were obtained from commercially available standard mixtures and minor SG were extracted from different food sources (oat bran, wheat bran, pumpkin seeds, melon, rapeseeds, and potato peel). ESI-MS/MS spectra of 15 FS were assessed and fragment ions reflective of structural features were identified and rationalized. Subsequently, 14 SG were identified at four different levels, while relative retention times from chromatographic separation and spectral features of FS served to identify five SG. Spectral data from FS were directly transferable to SG when analyzed as aglycone ions as shown by similarity scores while SG were characterized by shorter retention times in reverse phase chromatography and the additional analysis as sodiated adduct confirmed their glycosidic nature. Moreover, we report for the first time the occurrence of 24-methylenecholesterol and a 4-monomethyl sterol as glycosidic conjugates in higher plants. The presented data will serve as a valuable tool for SG profiling of foods by facilitating their identification.

## Introduction

**Figure 1:**
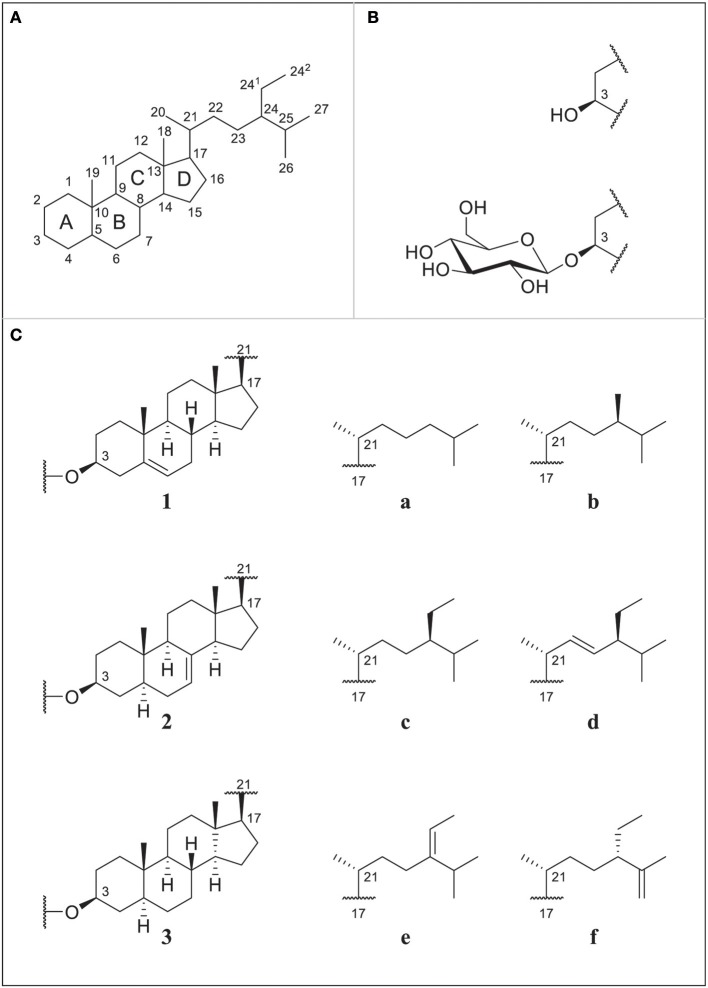
**(A)** Numbering of sterol backbone and labeling of condensed rings for phytosterols proposed by IUPAC 1989; **(B)** free sterols (FS) substituted at C3 with a hydroxyl group or steryl glucosides (SG) substituted at C3 with a glucose unit; **(C)** selection of different ring structures (1–3) and side chains (a–f); cholesterol (1a), campesterol (1b), sitosterol (1c), stigmasterol (1d), Δ^5^-avenasterol (1e), Δ^7^-campesterol (2b), Δ^7^-stigmastenol (2c), spinasterol (2d), Δ^7^-avenasterol (2e), poriferasta-7,25-dienol (2f), cholestanol (3a), campestanol (3b), and sitostanol (3c).

Dietary phytosterols ubiquitously occur in plant-based foods. Acclaimed for their various bioactive properties, including cholesterol-lowering effects, they are gaining much attention in the food industry. Phytosterols occur as free sterols (FS), steryl esters (SE), and glycosylated conjugates comprised of steryl glucosides (SG) and acylated steryl glucosides (ASG). Within this report, the focus lies on SG and their unbound counterpart as FS. Numerous compounds exist due to variations in the position and number of double bonds, differences in the side chain length, and the occurrence of stereoisomers (Figure 1). Unlike for FS, sterols with methylation at C4 have not been reported to occur as SG in higher plants (Wojciechowski, 1991; Potocka and Zirnowski, 2008).

Specific sterol profiles characteristic to certain plant families have been identified showing that a broad range of minor sterols occurs as free sterols or glycosylated conjugates (Nyström et al., 2012). While brassicaterol in either free or glycosylated form is characteristic to plants from the *Brassicaceae* family, Δ^7^-sterols are mostly found in *Cucurbitaceae* and *Amaranthaceae* plants (Akihisa et al., 1986; Xu et al., 1986; Nyström et al., 2012; Münger et al., 2015). Free or glycosylated Δ^5^-avenasterol is enriched in oat products and stanols are found at highest levels in whole wheat flour (Phillips et al., 2005; Nyström et al., 2012; Oppliger et al., 2014).

Determining the composition of sterol profiles is important as physiological and pharmacological properties such as intestinal absorption, metabolic fate, and bioactivity may be influenced by the sterol structure as was observed with FS (Ling and Jones, 1995) emphasizing the necessity for full structural knowledge. In case of SG, bioactive properties of glycosylated sterols, such as the reduction of intestinal cholesterol absorption and modulation of immune system, have been investigated with a primary focus on sitosteryl glucoside (Tateo et al., 1994; Bouic et al., 1996; Lee et al., 2007; Lin et al., 2009; Kim et al., 2013; Chávez-Santoscoy et al., 2014; Yang and An, 2014). To effectively study structure-activity relationships also for SG in the future, it is important to know the correct SG profiles of plants and to find sources for various SG including minor species. However, this requires the reliable identification and differentiation of SG species.

Due to analytical constraints, SG are less studied than other sterol species. SG profiles and amounts are generally determined by gas chromatography (GC) after acid hydrolysis and subsequent derivatization of released FS. Mass spectrometry (MS) has been acknowledged as a useful tool for revealing the identities of sterols in complex mixtures, with GC-MS/MS spectra of FS using electron impact ionization (EI) being widely described (Rahier and Benveniste, 1989; Goad and Akihisa, 1997). As a major drawback, acid hydrolysis induces isomerization of sterols with an ethylidene side chain as well as Δ^7^-sterols resulting in artifact formation and therefore preventing the determination of correct SG profiles (Kesselmeier et al., 1985; Kamal-Eldin et al., 1998; Phillips et al., 2005; Münger et al., 2015). Alternatively, SG can be hydrolyzed enzymatically prior to GC analysis to avoid isomerization (Kesselmeier et al., 1985; Münger and Nyström, 2014). The direct derivatization of intact SG has also been applied (Phillips et al., 2005; Gomez-Coca et al., 2012; del Rio et al., 2013; Prinsen et al., 2014).

Liquid chromatography (LC) methods offer the possibility to analyze sterols without prior derivatization (Breinhölder et al., 2002). Using MS for detection, co-eluting compounds can be differentiated based on their mass-to-charge ratio (*m/z*). Among phytosterols, MS/MS spectra have been obtained from the better studied FS as [FS-H_2_O+H]^+^ with atmospheric pressure chemical ionization (APCI) (Igarashi et al., 2011; Mo et al., 2013). Due to their higher polarity, electrospray ionization (ESI) provides a convenient mode of ionization for non-derivatized SG (Schrick et al., 2012; Oppliger et al., 2014). Tandem MS, which is based on collision induced dissociation (CID) of selected precursor ions, allows for further acquisition of MS/MS spectra that depend on the structure of the analyte, and provide an informative pattern of fragment ions. To the best of our knowledge, experimental spectral MS/MS data of native SG is not available yet, with the exception of sitosteryl glucoside acquired with APCI (Millán et al., 2016). Structural elucidation by LC-MS is not yet as straightforward as with GC-MS, mainly due to the lack of spectral libraries and simple fragmentation interpretation rules in general (Leonards et al., 2011). Additionally, commercial standards of SG are only available for the three most abundant species found in our diet (β-sitosteryl, campesteryl, and stigmasteryl glucoside) as well as cholesteryl glucoside, which occurs at low levels in certain plants such as *Solanaceae* (Münger and Nyström, 2014).

In order to fill this evident lack of spectral MS data from glycosylated sterols, this study aimed at generating ESI-MS/MS spectra of SG in order to facilitate identification in future profiling studies that are not restricted to only the most abundant species. Due to the limited number of available SG standards, the ESI-MS/MS spectra of FS standards, of which numerous compounds are commercially available, were first investigated. When selecting the aglycone ion of SG [SG-Glc+H]^+^ as the precursor ion, ESI-MS/MS spectra are identical with spectra of ionized FS as [FS-H_2_O+H]^+^ due to the common intermediate. Utilizing spectral data from FS, simple fragmentation rules could be deduced, enabling differentiation between isomeric forms and identification of SG, for which standards are unavailable. The use of the proposed LC-MS/MS approach resulted in the discovery of two sterols that have never been detected before as SG in higher plants.

## Materials and methods

### Materials

A commercially available SG mixture (purity >98%) consisting of approximately 56% sitosteryl glucoside, 25% campesteryl glucoside, 18% stigmasteryl glucoside, and 1% Δ^5^-avenasteryl glucoside (composition according to supplier information) was purchased from Matreya LLC (Pleasant Gap, USA), and was annotated as SG standard mixture in the following sections. Cholesteryl β-D-glucoside (≥97%) was obtained from Sigma-Aldrich (Buchs, Switzerland). Desmosterol (85%), 7-dehydrocholesterol (96.4%), cholestanol (98%), coprostanol (98%), ergosterol (95%), lanosterol (reagent grade), and stigmasterol (98%) were obtained from LGC Standards (Middlesex, UK), brassicasterol (97.5%) and 24-methylenecholesterol (+/−95%) from Research Plus (Barnegat, USA), campesterol (98%), sitosterol (98%), spinasterol (97%), stigmasterol (98%) from Toronto Research Chemicals (Toronto, Canada), and cholesterol (≥99%) from Sigma-Aldrich (Buchs, Switzerland).

UPLC-MS grade methanol was from Fisher Scientific AG (Reinach, Switzerland) and formic acid (LC-MS Ultra) from Sigma-Aldrich (Buchs, Switzerland). Purified water was prepared by a Milli-Q® purification system (Synergy UV, Millipore, USA). Hexane and isopropanol used for sample preparation were of HPLC grade (Sigma, Buchs, Switzerland). Leucine enkephaline was obtained from Waters (Milford, USA). Foods used for SG extraction were purchased in local grocery stores.

### Preparation of SG and FS standard solutions

Stock solutions of the SG standard mixture (0.2 mg/mL) and of cholesteryl glucoside (0.2 mg/mL) were prepared in 100% methanol. For FS, stock solutions at a concentration of 0.2 mg/mL dissolved in 2:98 v/v chloroform/methanol were prepared by first adding chloroform in order to guarantee full dissolution of FS. In case of sitostanol, a stock solution in 100% ethanol was used (0.1 mg/mL). After dilution of the stock solution with methanol, FS standards were injected at a final concentration of 0.02 mg/mL, SG standard solution at 0.04 mg/mL for minor SG and 0.005 mg /mL for major SG, and cholesteryl glucoside at 0.003 mg/mL.

### Extraction of sterols from foods

Melon (*Cucumis melo reticulatus*, only flesh) and potato peel (*Solanum tuberonum*, variety Agria) were dried and ground to a fine powder, whereas rapeseeds (*Brassica napus*), oat bran (*Avena sativa*), pumpkin seeds (*Cucurbita pepo*, raw), and wheat bran (*Triticum durum*) were directly ground. All foods were stored at −18°C. Food samples were subjected to accelerated solvent extraction (ASE 350 Dionex, Thermo Fisher Scientific, Reinach, Switzerland) and the SG fraction was isolated from the total lipid extract using solid phase extraction (SPE), for which the protocol has been published in detail elsewhere (Nyström et al., 2012). In short, total lipids were extracted from sample material (2 g of melon, potato peel, wheat bran and oat bran; 1 g of rapeseeds and pumpkin seeds) with acetone using ASE and the SG fraction was collected by eluting with 85:15 hexane:isopropanol from diol cartridges (GLScience, InertSep®, 500 mg, 3 mL). For oat bran, the fraction containing FS (97:3 hexane:isopropanol) was also collected and further processed. All samples were dried in a rotating evaporator under vacuum and residues were reconstituted in 5 mL methanol.

### UPLC-MS/MS analysis of FS and SG using electrospray ionization (ESI)

FS standards, SG standards and sterols extracted from food samples described above were injected into an ACQUITY Ultra-performance LC (UPLC) system (Waters, Milford, USA) using partial loop needle overfill mode (7.5 μl) and were separated on an UPLC ACQUITY BEH C18 column (50 × 2.1 mm; particle size, 1.7 μm, Waters, Milford, USA) by a water/methanol gradient elution with 0.1% formic acid as eluent additive at 40°C following a protocol published elsewhere (Oppliger et al., 2014). The UPLC was interfaced to a QTOF-MS system (Synapt G2, Waters, Milford, USA), which was operated in resolution mode using ESI in positive mode and was equipped with MassLynx acquisition software V4.1 (Waters, Milford, USA). For optimal mass accuracy, internal correction was performed during each run with the aid of leucine enkephaline (*m/z* 556.277) as lock-mass compound (well-defined ion with known elemental composition, Holcapek et al., 2012).

ESI was used to ionize all sterols within this study, as it was previously shown to be superior to APCI with regard to ion intensity in case of SG, when the intact molecule being investigated included the sugar moiety (Oppliger et al., 2014). Therefore, ESI was also applied to FS to ensure consecutive comparability with SG spectra even though APCI is the method of choice for FS because of higher ion intensity and consistency in ion formation (Baila-Rueda et al., 2013; Gachumi and El-Aneed, 2017).

MS tuning parameters were optimized aiming at the formation of aglycone ions [SG-Glc+H]^+^, since sodiated adducts [SG+Na]^+^ previously reported were too stable to undergo fragmentation (Oppliger et al., 2014). The formation of aglycone ions from SG [SG-Glc+H]^+^ is a result of glycosidic bond cleavage during in-source fragmentation and has been observed before in several publications using ESI or APCI (Rozenberg et al., 2003; Rudell et al., 2011; Wewer et al., 2011; Oppliger et al., 2014). Identification of compounds based on their aglycone ions formed by in-source fragmentation has been established also for other glycosidic compounds such as flavonoid glycoconjugates (Abrankó and Szilvassy, 2015).

Under the conditions applied, the intensity of [SG-Glc+H]^+^ was higher than of [SG+Na]^+^; however both ion species occurred. Aglycone ions [SG-Glc+H]^+^ were further selected as precursor ions for SG and were subjected to CID resulting in the production of fragment ions. FS generated high intensity ions as [FS-H_2_O+H]^+^, which were selected as precursor ions for FS analysis. Conditions were as follows: capillary voltage, 3.0 kV; sampling cone voltage, 23 V; extraction cone, 4.0 V; source temperature, 130°C, desolvation temperature, 600°C; cone gas flow, 35 L/h; and desolvation gas flow, 850 L/h. Low mass (LM) resolution was set to 15, which assured narrow *m/z* isolation of selected ions. Trap collision energy for CID was 25 V for all selected ions and the collision gas was argon (purity > 99.9%). MS/MS-spectra of stigmasterol, ergosterol, 24-methylenecholesterol, brassicasterol, and spinasterol were additionally acquired at 30 V. The MS acquisition method consisted of one full MS scan (50-1200 *m/z*, acquisition time: 0.0–13.0 min, scan time: 0.5 s, centroid data mode) and a MS/MS scan for selected precursor ions (scan time: 0.3 s, acquisition time: 6.0–10.0 min, centroid data mode). The *m/z* of [SG+Na]^+^ and [SG-Glc+H]^+^, the latter corresponding to *m/z* of the respective [FS-H_2_O+H]^+^, are displayed in Table 1. Relative retention times (RRT) of FS and SG were calculated with regard to cholesterol and cholesteryl glucoside, respectively. Overlayed extracted ion chromatograms for representation of the elution order were processed in Microsoft Excel 2016, whereas only peaks of interest were isolated by selecting limited time windows for each chromatogram.

**Table 1:** Nomenclature of sterol moieties and mass of free sterols (FS) and steryl glucosides (SG); grouped by isomers in order of increasing mass; mass-to-charge-ratios (*m/z*) are given for sodiated SG adducts [SG+Na]^+^, and the respective aglycone ions [SG-Glc+H]^+^, which share the same *m/z* as dehydrated ions of FS [FS-H_2_O+H]^+^.

**Common name**	**Systematic name**	**Mass of FS [Da]**	**Mass of SG^a^ [Da]**	***m/z* of [SG+Na]^+^**	***m/z* of [SG-Glc+H]^+^ and [FS- H_2_O+H]^+^**
Desmosterol 7-dehydrocholsterol	cholesta-5,24-dien-3β-ol cholesta-5,7-dien-3β-ol	384.339	546.392	569.382	367.336
Cholesterol	cholest-5-en-3β-ol	386.355	548.408	571.398	369.352
Cholestanol Coprostanol	5α-cholestan-3β-ol 5β-cholestan-3β-ol	388.371	550.423	573.413	371.368
Ergosterol	(22E)-ergosta-5,7,22-trien- 3β-ol	396.339	558.392	581.382	379.336
Brassicasterol 24-Methylene-cholesterol	(22E,24S)-24-methylcholesta-5,22-dien-3β-ol 24-methylidene-cholest-5-en-3β-ol	398.355	560.408	583.398	381.352
Campesterol Δ^7^-Campesterol	(24R)-24-methylcholest-5-en-3β-ol (24R)-24-methyl-5α-cholest-7-en-3β-ol	400.371	562.423	585.413	383.368
Campestanol	(24R)-24-methyl-5α-cholestan-3β-ol	402.386	564.439	587.429	385.383
Poriferasta-7,22,25-trienol	(22E,24S)-24-ethyl-5α-cholesta-7,22,25-trien-3β-ol	410.355	572.408	595.398	393.352
Stigmasterol Δ^5^-Avenasterol Spinasterol Δ^7^-Avenasterol Poriferasta-7,25-dienol	(22E,24S)-24-ethylcholesta-5,22-dien-3β-ol (24Z)-24-ethylidenecholest-5-en-3β-ol (22E,24S)-24-ethyl-5α-cholesta-7,22-dien-3β-ol (24Z)-24-ethylidene-5α-cholest-7-en-3β-ol (24S)-24-ethyl-5α-cholesta-7,25-dien-3β-ol	412.371	574.423	597.413	395.368
Sitosterol Δ^7^-Stigmastenol	(24R)-24-ethylcholest-5-en-3β-ol (24R)-24-ethyl-5α-cholest-7-en-3β-ol	414.386	576.439	599.429	397.383
Sitostanol	(24R)-24-ethyl-5α-cholestan-3β-ol	416.402	578.455	601.444	399.399
Citrostadienol Cycloartenol Lanosterol	(24Z)-4α-methyl-24-ethylidene-5α-cholest-7-en-3β-ol 4,4,14-trimethyl-9,19-cyclo-5α,9β-cholest-24-en-3β-ol 4,4,14-trimethyl-5α-cholesta-3β-ol	426.386	588.439	611.444	409.383

a *With β-D-glucopyranose as a sugar moiety*.

### Calculation of spectral similarity score

In order to compare MS/MS spectra obtained from SG and its corresponding FS or isomeric compounds, a spectral similarity score was calculated according to Equation (1) with $I_{m}^{1}$ and $I_{m}^{2}$ being intensities of an ion at *m/z* = m for the two spectra; a procedure that has been presented for ESI-MS/MS spectral comparison already elsewhere (Mulroney et al., 1995; Boulos and Nyström, 2016).

(1) $$similarity=\frac{\sum \sqrt{I_{m}^{1}I_{m}^{2}}}{\sqrt{\left(\sum I_{m}^{1}\right)\left(\sum I_{m}^{2}\right)}}$$

A score of 0 represents no spectral similarity, while a value of 1 stands for complete spectral similarity. A set of sterol characteristic ions were selected based on the ten most intense fragment ions in the MS/MS spectra of each FS analyzed within this study. A total of 39 fragment ions, as well as the respective precursor ion, were thus selected (see Table S1) and their absolute intensities were retrieved from the ESI-MS/MS spectra of the investigated SG and FS for further similarity calculation. Similarity was also calculated among all FS even if they were non-isomeric compounds. In this case, the precursor ion was excluded for the calculation. A similarity score higher than 0.99 indicated equal ESI-MS/MS spectra, as found within the current data when comparing the spectra of FS and corresponding SG, for which standard compounds were available.

### Definition of identification levels of SG

SG were identified at different levels for this spectral library. Level 1: spectral data and RRT were obtained from SG standards. Level 2: spectral data and RRT of FS were available and were used to confirm identity of SG. As previously published, elution order of FS on a reverse phase column was applicable to corresponding glycosides (Rozenberg et al., 2003). Level 3: *m/z* of the sodiated adduct of SG and previous knowledge about occurrence of minor species in specific food source were available and used for identification. The elution order of known dietary Δ^7^-steryl glucosides (Δ^7^-avenasterol glucoside, spinasterol glucoside, Δ^7^-stigmasterol glucoside, poriferasta-7,25-glucoside and poriferasta-7,22,25-glucoside) was deduced from the reports by Breinhölder et al. (2002) who used a C8 column for SG isolated from pumpkin seeds, and from Strobl (2004) who used a RP-HPLC C18 column, as well as previous knowledge about SG composition of pumpkin seeds and melon (Münger et al., 2015). Level 4: *m/z* of sodiated adduct of SG was linked to elemental composition, and thus, to possible SG species while previous knowledge was not available.

## Results and discussion

### Chromatographic separation and characterization of ESI-MS/MS spectra of FS

**Figure 2:**
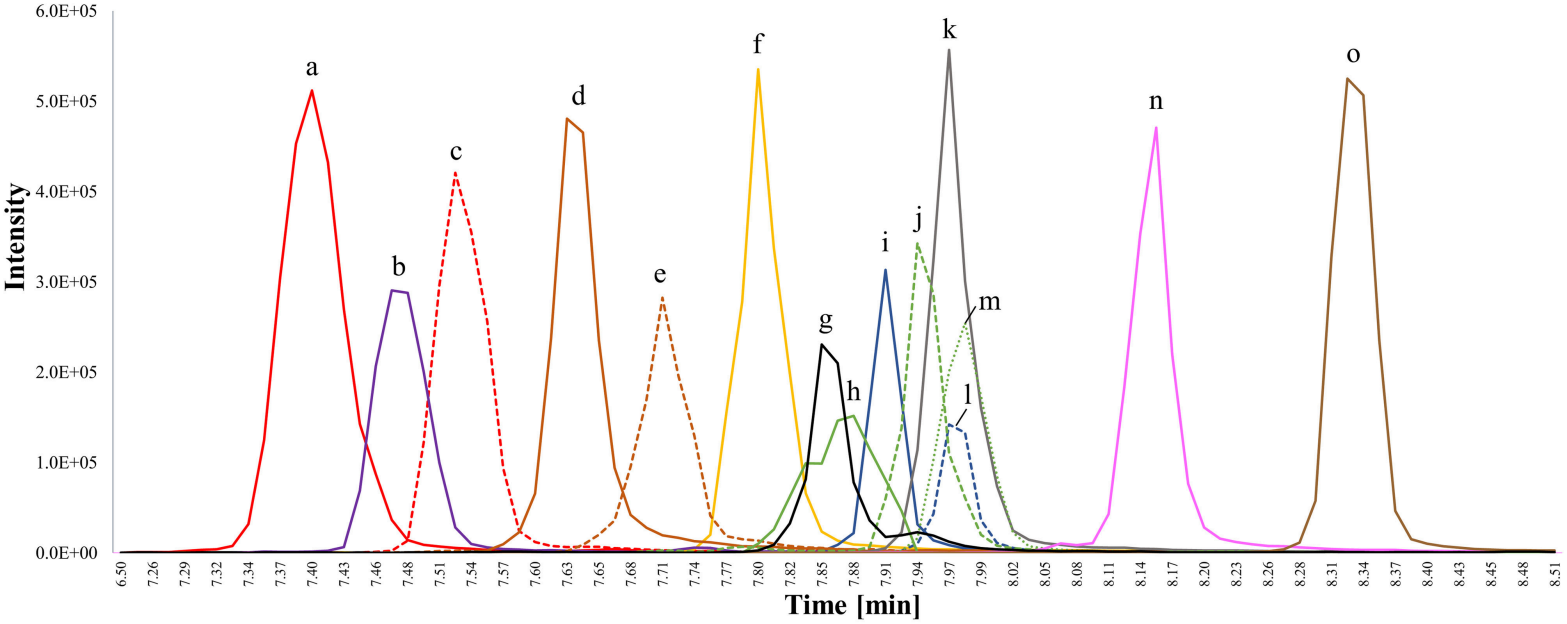
Overlay of extracted ion chromatograms (EIC) for chromatographic separation of free sterol (FS) standards detected as [FS-H_2_O+H]^+^ on a C18 UPLC column applying a methanol/water gradient with 0.1% formic acid as additive; isomeric compounds have the same color and are distinguished by different line patterns. (a) Desmosterol (*m/z* 367; C27; Δ^5^,Δ^24(25)^); (b) ergosterol (*m/z* 379; C28; Δ^5^,Δ^7^,Δ^22^); (c) 7-dehydrocholesterol (*m/z* 367; C27; Δ^5^,Δ^7^); (d) 24-methylenecholesterol (*m/z* 381; C28; Δ^5^,Δ^24(24^1^^)); (e) brassicasterol (*m/z* 381; C28; Δ^5^,Δ^22^); (f) cholesterol (*m/z* 369; C27, Δ^5^); (g) lanosterol (*m/z* 409; C30, Δ^8^); (h) Δ^5^-avenasterol extracted from oat (*m/z* 395; C29, Δ^5^,Δ^24(24^1^)^); (i) coprostanol (*m/z* 371; C27, stanol with β-configuration of hydrogen at C5); (j) spinasterol (*m/z* 395; C19; Δ^7^,Δ^22^); (k) campesterol (*m/z* 383; C28; Δ^5^); (l) cholestanol (*m/z* 371; C27, stanol with α-configuration of hydrogen at C5); (m) stigmasterol (*m/z* 369; C29; Δ^5^,Δ^22^); (n) sitosterol (*m/z* 397; C29; Δ^5^), and (o) sitostanol (*m/z* 399; C29; fully saturated). Accurate *m/z* are listed in Table 1 and peaks were extracted from chromatograms with a mass window of 0.01 Da.

FS eluted between 7.38 min (desmosterol) and 8.34 min (sitostanol) but complete baseline separation of all analyzed FS remained difficult on a C18 column even under gradient elution due to high structural similarity (Figure 2), which is a known limitation in sterol analysis (Moreau et al., 2018). RRT were not only obtained for phytosterols but also for sterols found in fungi (ergosterol) and animals (desmosterol, 7-dehydrocholesterol, coprostanol, cholestanol, lanosterol) in order to increase structural diversity among the analyzed standards (Table 2). Due to its relevant role in foods, Δ^5^-avenasterol was extracted from oat bran. Same elution order on a reverse phase C18 cartridge as reported here was observed by Baila-Rueda et al. (2013) for phytosterols (campesterol, stigmasterol, sitosterol, and sitostanol) and cholesterol and its precursors (desmosterol, lanosterol, and cholestanol) occurring in human serum. The present study extended the knowledge about the elution order to 15 sterols in total. Generally, increasing degree of unsaturation in the B-ring or in the side chain resulted in shorter retention time when comparing non-isomeric compounds such as sitosterol and sitostanol (Figure 2). The trend of unsaturation leading to lower retention on the C18-column is in agreement with the hydrophobicity being a function of saturation. Among the three isomers with *m/z* 395, Δ^5^-avenasterol (Δ^5^,Δ^24(24^1^^)) eluted first, followed by spinasterol (Δ^7^,Δ^22^) and its Δ^5^-form stigmasterol (Δ^5^,Δ^22^), though not fully baseline separated. Equally, 24-methylenecholesterol (Δ^5^,Δ^24(24^1^^)) showed shorter retention times than its corresponding Δ^5^,Δ^22^-sterol (brassicasterol) but they still overlapped. The two epimeric forms cholestanol (5α-configuration) and coprostanol (5β-configuration) eluted at different times even though they only differ in the configuration at C5. Among C27-sterols with two double bonds, desmosterol (Δ^5^,Δ^24(25)^) eluted prior to its isomer 7-dehydrocholesterol (Δ^5^,Δ^7^).

**Table 2:** Relative retention times (RRT) of free sterols (FS) on a C18 UPLC column with a methanol/water gradient.

**FS**	**RRT to cholesterol**
Desmosterol	0.95
Ergosterol	0.96
7-Dehydrocholesterol	0.97
24-Methylenecholesterol	0.97
Brassicasterol	0.99
Cholesterol	1.00
Δ^5^-Avenasterol^a^	1.00
Lanosterol	1.01
Coprostanol	1.01
Spinasterol	1.02
Cholestanol	1.02
Campesterol	1.02
Stigmasterol	1.02
Sitosterol	1.05
Sitostanol	1.07

a *Not obtained as pure standard compound but extracted from oat bran*.

For all FS analyzed in the present study, the ion [FS-H_2_O+H]^+^ was at sufficient intensity and ESI-MS/MS spectra at 25 V were obtained for all of them (complete overview as Figures S1–S15). Spectral similarity score among all FS ranged from 0.52 to 1.00 (Table S2) demonstrating that the similarity score based on a selection of characteristic fragment ions was able to differentiate between equal and differing fragment ion spectra. Similarity scores between stanols (cholestanol and sitostanol) and between sitosterol and campesterol as well as between stigmasterol and brassicasterol was higher than 0.99 pointing out that fragmentation of sterols only differing in alkylation at C24 resulted in identical fragmentation pattern and were exclusively differentiated by the *m/z* of the precursor ion.

**Figure 3:**
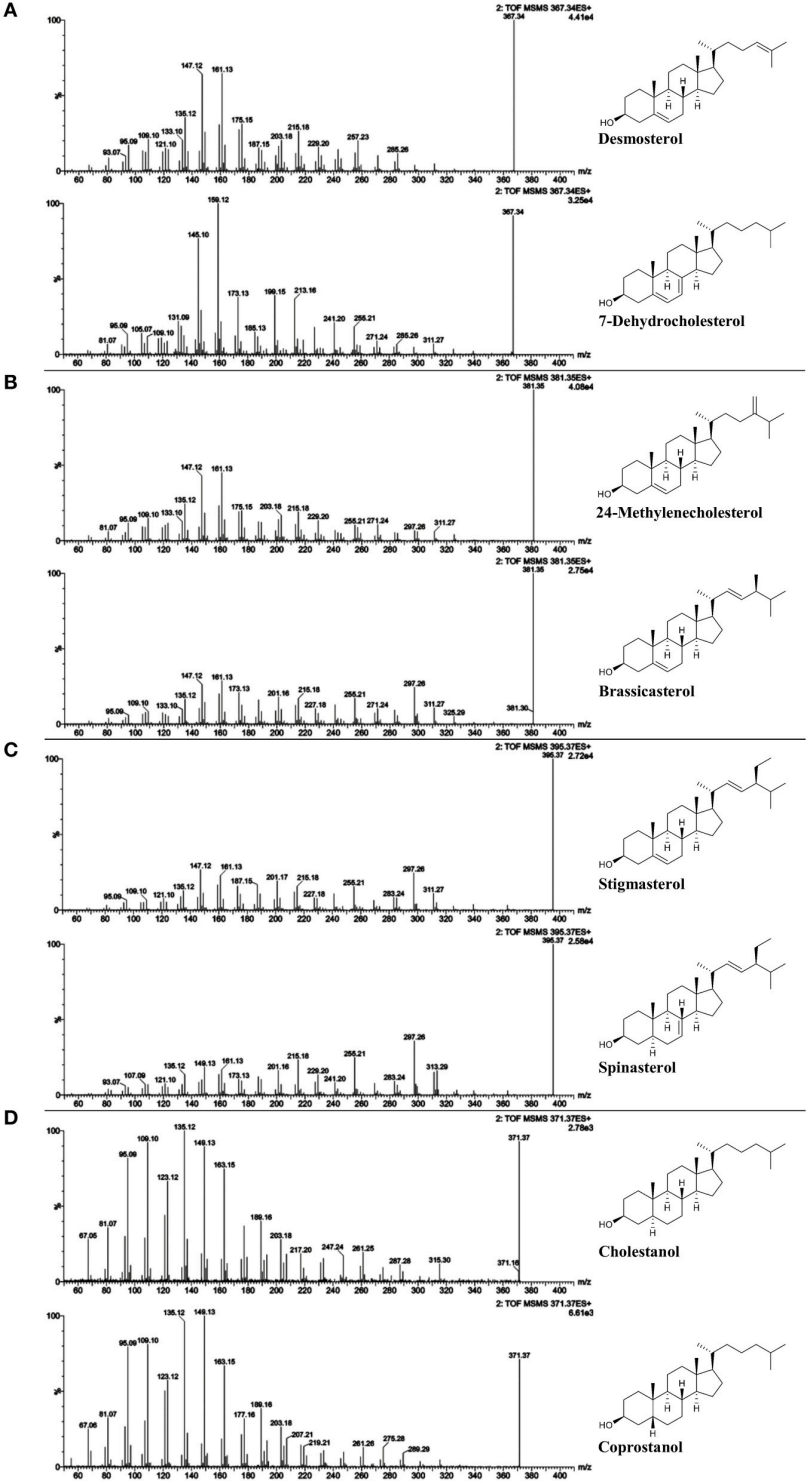
ESI-MS/MS spectra (25 V) obtained from isomeric free sterol (FS) standards as [FS-H_2_O+H]^+^; **(A)** desmosterol and 7-deyhdrosterol with *m/z* 367; **(B)** 24-methylenecholesterol and brassicasterol with *m/z* 381; **(C)** spinasterol and stigmasterol with *m/z* 395; and **(D)** cholestanol and coprostanol with *m/z* 371.

Among isomeric sterols, spectral similarity reached high scores (Table 3) demonstrating that good chromatographic resolution is crucial for their distinction. Full baseline separation was not achieved for all isomers but in all cases distinct double peaks were recognizable (Figure 2). The two epimers cholestanol (RRT 1.02) and coprostanol (RRT 1.01) were not distinguishable based on their spectral data (similarity score = 1.00) whereas for stigmasterol (RRT 1.02) and spinasterol (RRT 1.02) the similarity score was 0.98, for brassicasterol (RRT 0.99) and 24-methylenecholesterol (RRT 0.97) it was 0.98, and for desmosterol (RRT 0.95) and 7-dehydrocholesterol (RRT 0.97) it was 0.90. Between these isomers, minor differences within the fragmentation spectra were observed (Figure 3), which are discussed in more detail below and expanded to all analyzed FS.

**Table 3:** Spectral similarity score of isomeric free sterols (FS) and their structural differences.

**Isomeric FS**	**Spectral similarity score**	**Structural differences**
Cholestanol/coprostanol	1.00	α-C5/β-C5
Stigmasterol/spinasterol	0.98	Δ^5^,Δ^22^/Δ^7^,Δ^22^
Desmosterol/7-dehydrocholesterol	0.90	Δ^5^,Δ^24(25)^/Δ^5^,Δ^7^
24-Methylenecholesterol/brassicasterol	0.98	Δ^5^,Δ^24(24^1^^)/Δ^5^,Δ^22^

#### Intensity of precursor ion

Unlike sterols with a fully saturated side chain, FS with unsaturated side chains exhibited the precursor ion as dominant base peak at 25 V collision energy as shown in case of brassicasterol, 24-metyhlenecholesterol, stigmasterol, and spinasterol (Figures 3B,C). In case of FS with unsaturation at position C22 or C24(24^1^), 30 V resulted in MS/MS-spectra with fragment ions as base peaks while the precursor ions remained detectable (Figures S3–S5, S10, S12, part b). LC-MS/MS spectra of sterols have been mostly investigated on FS as carbocations after dehydration [FS-H_2_O+H]^+^ using APCI. As reported by Igarashi et al. (2011), higher collision energy had to be applied for the formation of dominant fragment ions relative to the precursor ion in case of stigmasterol when compared to campesterol and sitosterol.

#### C-ring fragmentation

The ions formed by the fragmentation within the C-ring were generally at highest intensity among all fragments at 25 V and delivered information about the degree of unsaturation within the B-ring (Figure 3, Figures S1–S15). Scission between C9/C11 and C8/14 led to *m/z* 147 with higher relative abundance than *m/z* 149 and *m/z* 145 for Δ^5^-sterols. In contrast, higher intensity of *m/z* 149 than both *m/z* 147 and *m/z* 145 was characteristic for Δ^7^-sterols, stanols and lanosterol, whereas for Δ^5^,Δ^7^-sterols, *m/z* 145 was higher than *m/z* 147 and *m/z* 149. In addition, scission between C11/C12 and C8/C14 resulted in *m/z* 161 exceeding *m/z* 163 in intensity for Δ^5^- and Δ^7^-sterols while for Δ^5^,Δ^7^-sterols and for stanols the predominant species were *m/z* 159 and *m/z* 163, respectively. As reported, the most abundant fragment ion was *m/z* 147 for cholesterol, campesterol, stigmasterol, and sitosterol when using APCI (Igarashi et al., 2011), which was also found in the current study. In addition, the authors observed that *m/z* 145 was the second most abundant fragment ion for sterols with two double bonds in the B-ring (7-dehydrocholesterol and ergosterol) (Igarashi et al. (2011), which could be confirmed in this study.

**Figure 4:**
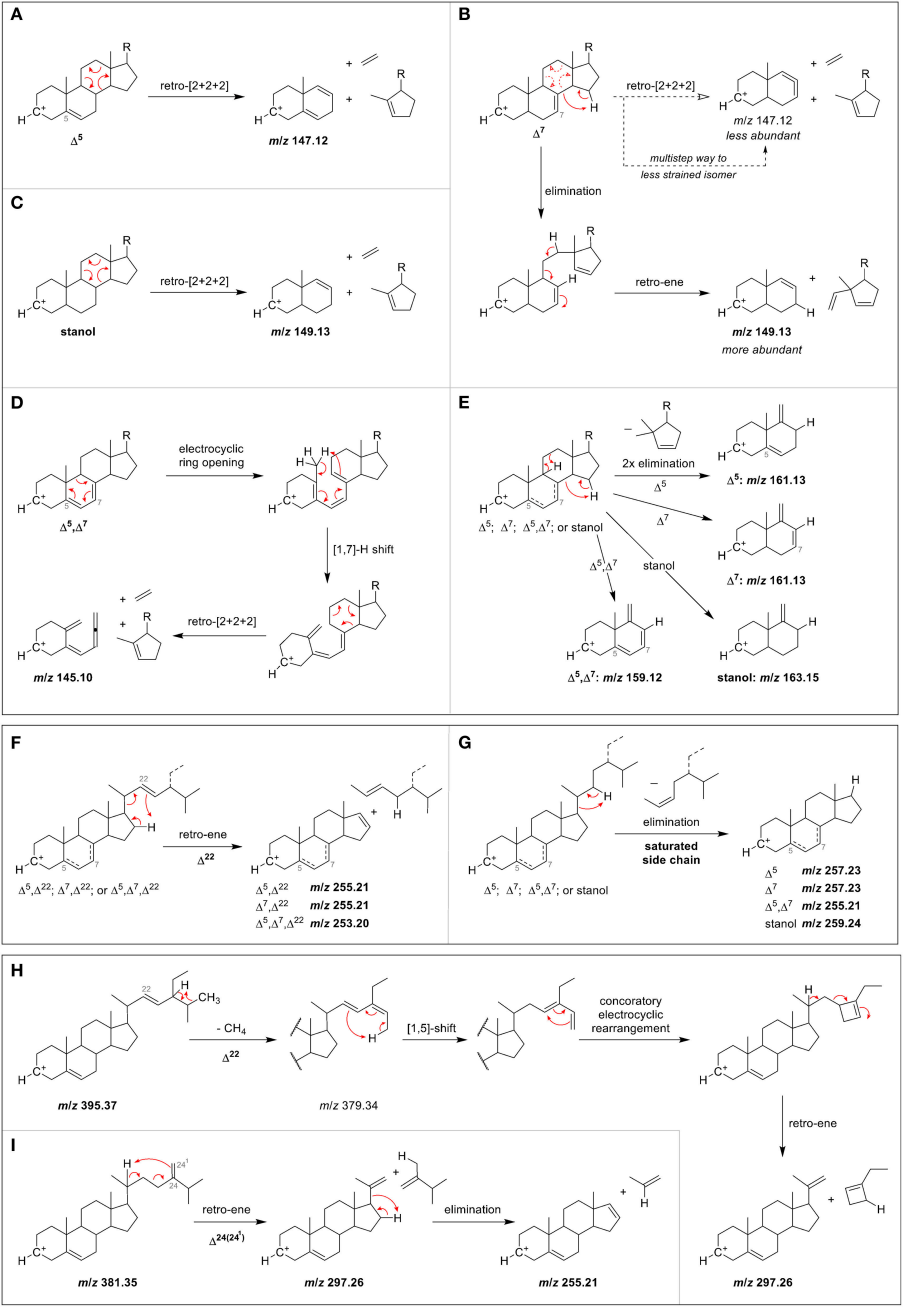
Proposed MS/MS fragmentation pathways for the formation of characteristic ions resulting from C-ring fragmentation in case of **(A)** Δ^5^-sterol, **(B)** Δ^7^-sterol, **(C)** stanol, and **(D)** Δ^5^,Δ^7^-sterol, as well as **(E)** partial C-ring fragmentation. Also shown are proposed mechanisms for side chain loss in case of **(F)** any Δ^22^-sterols, **(G)** any sterols or stanols with saturated side chain, and partial side chain loss in the case of **(H)** a Δ^5^,Δ^22^-sterol, and **(I)** a Δ^5^,Δ^24(24^1^)^-sterol.

The fragment *m*/*z* 147 may be rationalized by a charge-remote [2+2+2]-cycloreversion reaction of the C-ring as shown in the proposed fragmentation pathway for Δ^5^-sterols (Figure 4A) (Demarque et al., 2016). The formation of the fragment ion *m*/*z* 149, which was characteristic for Δ^7^-sterols and stanols may be rationalized by an elimination–retro-ene sequence for the former, and a direct [2+2+2]-cycloreversion for the latter (Figures 4B,C). The reason why *m*/*z* 147 is not more dominant for Δ^7^- compared to Δ^5^-sterols lies in the highly strained nature of the cyclic allene that would be the result of a direct [2+2+2]-cycloreversion for Δ^7^-sterols due to the position of the double bond, rendering this pathway less favorable (or forcing a different, less yielding multi-step pathway to form the *m*/*z* 147 fragment). The formation of *m*/*z* 145 in Δ^5^,Δ^7^-sterols, on the other hand, is proposed to involve an electrocyclic ring opening of the doubly unsaturated B-ring to the corresponding triene with possible [1,7] sigmatropic hydride-shift prior to retro-[2+2+2]-reaction of the C-ring, hence allowing a straightforward pathway of C-ring cleavage despite the Δ^7^ double bond (Figure 4D). The formation of the fragment class *m*/*z* 163, 161, and 159 resulting from two elimination reactions is simply governed by the number of double bonds in stanols, Δ^5^ ^or^ ^7^-, and Δ^5^,Δ^7^-sterols, respectively (Figure 4E).

#### Side chain loss

The loss of the side chain led to different fragment ions (*m/z* 253, 255, 257, 259) depending on the degree of unsaturation of the side chain as well as of the B-ring. *m/z* 253 as predominant species was observed only in case of Δ^5^,Δ^7^,Δ^22^ (multiple ring unsaturation and side chain unsaturation at position C22). *m/z* 255 occurred at higher intensities than *m/z* 257 and *m/z* 253 for Δ^5^- and Δ^7^-sterols that contained a double bond in the side chain at either position C22 (i.e., brassicasterol, stigmasterol and spinasterol, Figures 3B,C) or C24(24^1^) as in 24-methylenecholesterol (Figure 3B). Loss of side chain among Δ^5^,Δ^7^-sterols with a saturated side chain resulted in *m/z* 255, too, as shown for 7-dehydrocholesterol (Figure 3A). Opposed to that, Δ^5^-desmethylsterols with a saturated side chain as in sitosterol (Figure S13) or one double bond at position C24(25) as in desmosterol (Figure 3A) were characterized by higher *m/z* 257 than *m/z* 255 and *m/z* 253. The position of the double bond at either C24(24^1^) or C24(25) thus affected the ion formed by the loss of the side chain. Among stanols, sitostanol was characterized by *m/z* 259 (Figure S14) together with cholestanol and coprostanol (Figure 3D). The presence of the more abundant *m/z* 259 and *m/z* 261 than the ion formed by side chain loss in the spectra of cholesterol (Figure S6) and cholestanol (Figure S9), respectively, may be originating from other fragmentation pathways than loss of the side chain.

Proposed fragmentation pathways suggest the involvement of a retro-ene reaction for sterols with one C22 double bond in the side chain, leading to *m/z* 255 for Δ^5^,Δ^22^- and Δ^7^,Δ^22^-sterols, and *m*/*z* 253 for Δ^5^,Δ^7^,Δ^22^-sterols (Figure 4F). On the other hand, saturated side chains are cleaved by elimination without introduction of a double bond on the D-ring, making the resulting fragment purely governed by the number of double bonds in the B-ring, with *m*/*z* 259, *m*/*z* 257, and *m*/*z* 255 for zero, one, and two double bonds, respectively (Figure 4G). For sterols with double bond on C24(24^1^), fragmentation occurs over a partial side chain loss intermediate through a retro-ene reaction that is sterically hindered for Δ^24(25)^-sterols, hence explaining why the latter has a less pronounced *m*/*z* 255 ion (Figure S16).

#### Partial side chain fragmentation

The fragment ion *m/z* 297 was among the three highest intensity ions only for Δ^5^- and Δ^7^-desmethylsterols with a double bond at C22 as in stigmasterol, spinasterol (Figure 3C), and brassicasterol (Figure 3B). For Δ^5^,Δ^24(25)^- and Δ^5^,Δ^24(24^1^)^-sterols as in desmosterol (Figure 3A) and 24-methylenecholesterol (Figure 3B), respectively, this fragment ion was not as pronounced while it was virtually absent in the MS/MS spectra of sterols with fully saturated side chains. The fragment ion with *m/z* 297 was assigned to a multi-step reaction involving demethylation, conrotatory electrocyclic rearrangement and retro-ene reaction in case of a Δ^5^,Δ^22^-sterol (Figure 4H) while for a Δ^5^,Δ^24(24^1^)^-sterol this fragment ion may be formed by direct retro-ene reaction (Figure 4I). Within a saturated side chain, a retro-ene reaction is not possible, hence explaining the absence of this fragment ion in the spectra of sterols with saturated side chains.

Rozenberg et al. (2003) presented MS/MS spectra of isomeric stigmasterol, Δ^7^-avenasterol, and Δ^5^-avenasterol using APCI. Differentiation of stigmasterol from the two others was possible by the presence of a more dominant ion with *m/z* 297. Mo et al. (2013) noted that *m/z* 297 appeared in the APCI-MS/MS spectra of brassicasterol and stigmasterol (both with Δ^22^-bond) due to partial elimination of the side chain. These findings confirm our observation that unsaturated side chains are more likely to undergo partial cleavage of the side chain yielding *m/z* 297.

### Chromatographic separation and characterization of ESI-MS/SM spectra of SG

**Figure 5:**
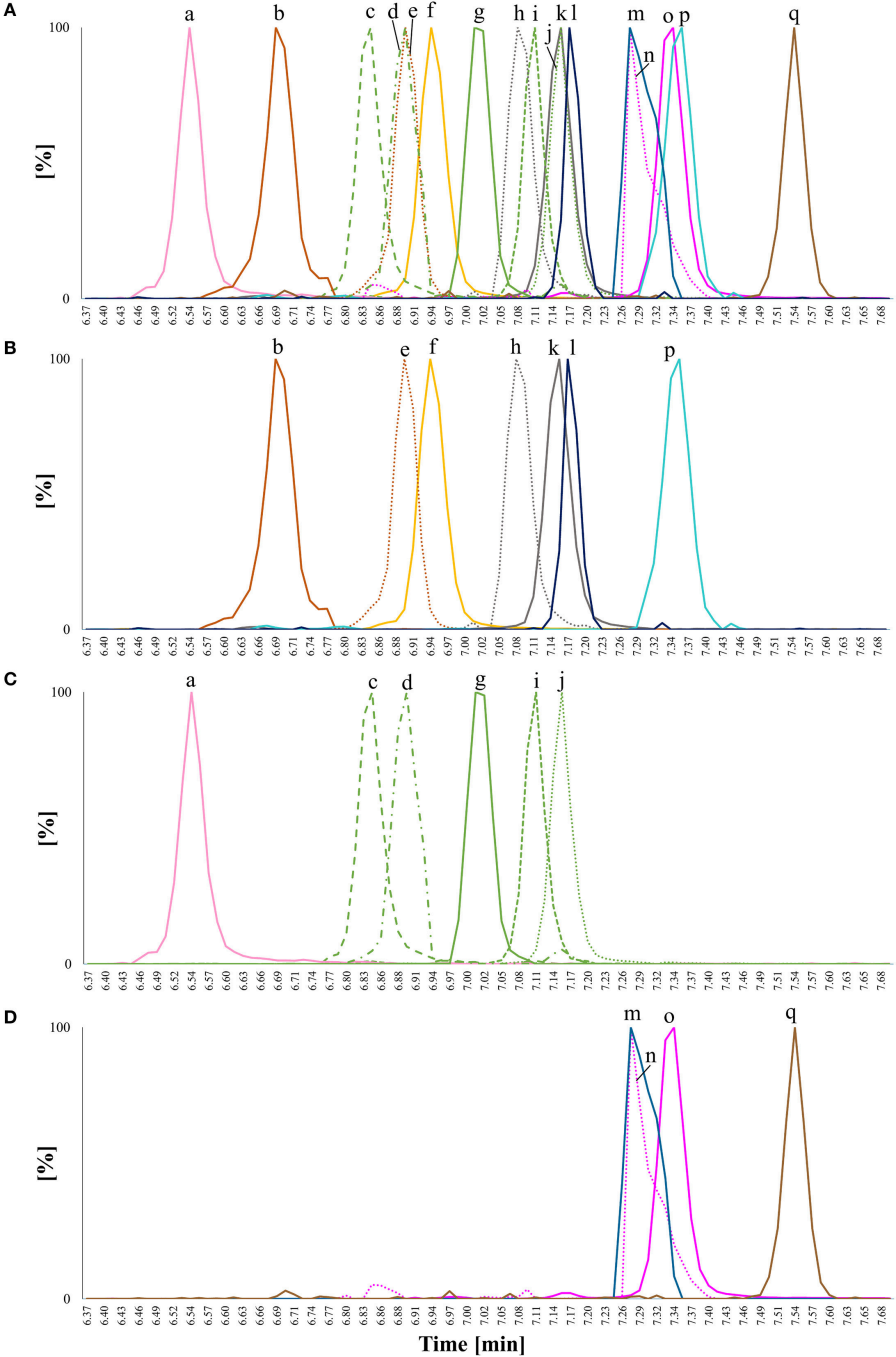
Overlay of extracted ion chromatograms (EIC) for chromatographic separation of steryl glucosides (SG) from standards or extracted from foods detected as [SG+Na]^+^ on a C18 UPLC column applying a methanol/water gradient with 0.1% formic acid as additive; isomeric compounds have the same color and are distinguished by different line patterns; **(A)** EIC of all analyzed SG in one plot; **(B)** EIC of SG with *m/z* 571 - *m/z* 587; **(C)** EIC of SG with *m/z* 595 - *m/z* 597, and **(D)** EIC of SG with *m/z* 599 - *m/z* 611; (a) poriferasta-7,22,25-dienyl glucoside (*m/z* 595) from pumpkin seeds; (b) 24-methylenecholesteryl glucoside (*m/z* 583) from rapeseeds; (c) poriferasta-7,25-dienyl glucoside (*m/z* 597) from pumpkin seeds; (d) Δ^7^-avenasteryl glucoside (*m/z* 597) from SG standard mixture; (e) brassicasteryl glucoside (*m/z* 583) from rapeseeds; (f) cholesteryl glucoside (*m/z* 571) from a standard; (g) Δ^5^-avenasteryl glucoside (*m/z* 597) from oat bran; (h) Δ^7^-campesteryl glucoside (*m/z* 585) from melon; (i) spinasteryl glucoside (*m/z* 597) from pumpkin seeds; (j) stigmasteryl glucoside (*m/z* 597) from SG standard mixture; (k) campesteryl glucoside (*m/z* 585) from SG standard mixture; (l) cholestanyl glucoside (*m/z* 573) from potato peel; (m) citrostadienyl glucoside (*m/z* 611) from potato peel; (n) Δ^7^-stigmastenyl glucoside (*m/z* 599) from melon; (o) sitosteryl glucoside (*m/z* 599) from SG standard mixture; (p) campestanyl glucoside (*m/z* 587) from wheat bran and (q) sitostanyl glucoside (*m/z* 601) from wheat bran. Accurate *m/z* are listed in Table 1 and peaks were extracted from chromatograms with a mass window of 0.01 Da. Only peaks of interest were isolated.

The difference in elution time between sterols as SG and FS on the C18 column using a methanol/water gradient was 0.88 min (53 s), with SG having shorter retention times than FS. The RRT obtained from SG (Table 4) compared to RRT from the sterols as FS (Table 2) differed only minimally, while elution order were completely identical suggesting that RRT and elution order obtained from FS (Figure 2) were directly transferable to SG (Figure 5). Small differences in RRT compared to FS standards may derive from concentration differences as minor SG occur at reasonably low levels in foods. ESI-MS/MS spectra were obtained for 14 SG species, out of which five SG were identified based on spectral and chromatographic data obtained from previous FS analysis (level 2 identification).

**Table 4:** Relative retention times (RRT) of steryl glucosides (SG) on a C18 UPLC column and their level of identification in the frame of the present study.

**SG**	**RRT to cholesteryl glucoside**	**Level of identification^a^**	**MS/MS spectra obtained^b^**	**Source**
Poriferasta-7,22,25-trienyl glucoside	0.94	3	Yes	Pumpkin seeds
24-Methylenecholesteryl glucoside	0.97	2	Yes	Rapeseeds
Poriferasta-7,25-dienyl glucoside	0.99	3	Yes	Pumpkin seeds
Brassicasteryl glucoside	0.99	2	Yes	Rapeseeds
Cholesteryl glucoside	1.00	1	Yes	Standard
Δ^7^-Avenasteryl glucoside	1.00	3	No	SG standard mixture
Δ^5^-Avenasteryl glucoside	1.00	2^c^	Yes	Oat bran
Δ^7^-Campesteryl glucoside	1.02	3	Yes	Melon
Cholestanyl glucoside	1.02	3	No	Potato peel
Spinasteryl glucoside	1.03	2	Yes	Pumpkin seeds
Campesteryl glucoside	1.03	1	Yes	SG standard mixture
Stigmasteryl glucoside	1.03	1	Yes	SG standard mixture
Citrostadienyl glucoside	1.04	4^d^	Yes	Potato peel
Campestanyl glucoside	1.05	3	no	Wheat bran
Δ^7^-Stigmastenyl glucoside	1.05	3	Yes	Melon
Sitosteryl glucoside	1.05	1	Yes	SG standard mixture
Sitostanyl glucoside	1.08	2	Yes	Wheat bran

a *See experimental section for the definition of the 4 levels*.

b *Only if ion intensity was sufficient (>1x10^4^) in respective food source, MS/MS-spectra could be obtained*.

c *FS standard was extracted from food source*.

d *Identification further confirmed by GC-MS experiments*.

#### Level 1 identified SG—sitosteryl glucoside, campesteryl glucoside, stigmasteryl glucoside, and cholesteryl glucoside

**Figure 6:**
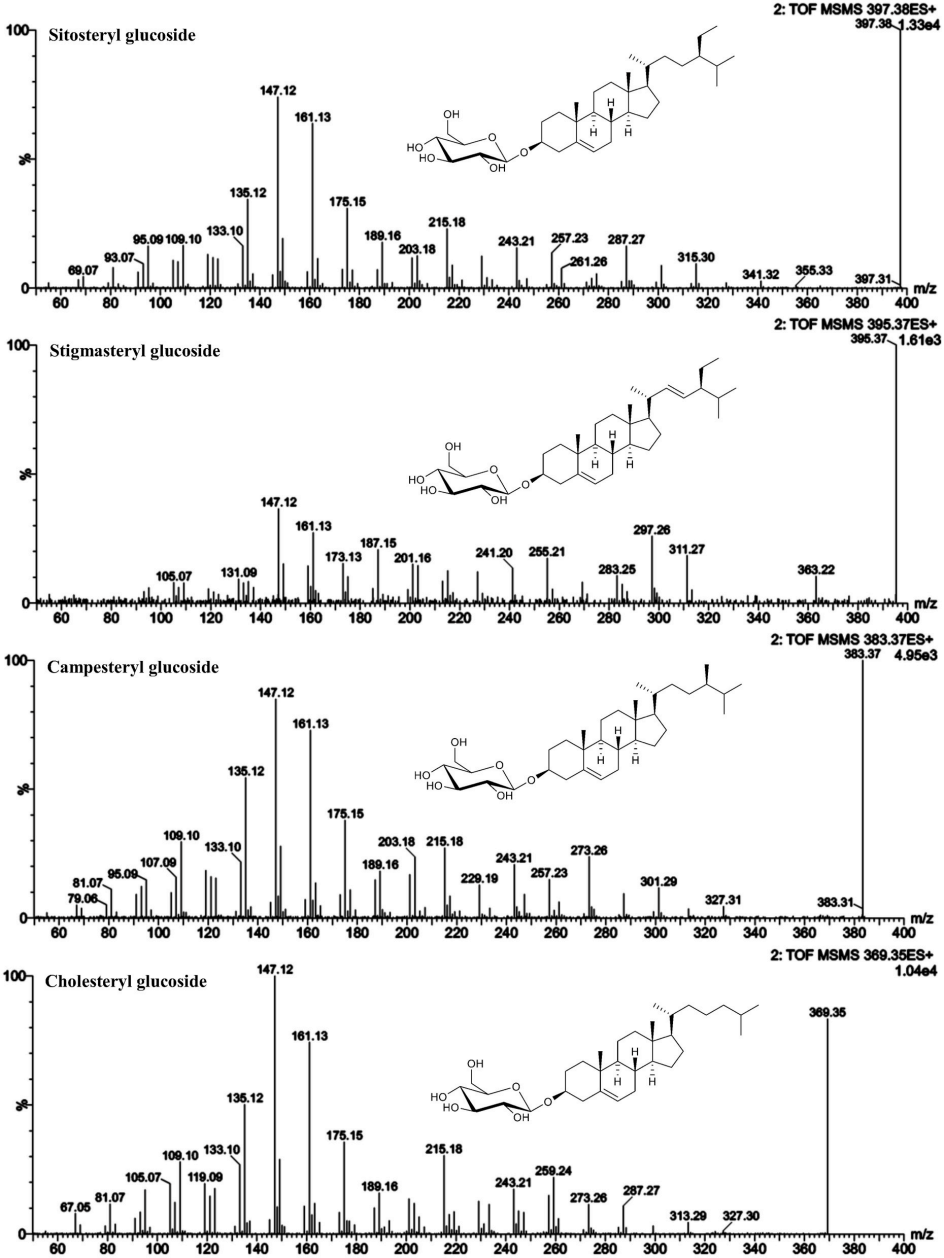
ESI-MS/MS spectra (25 V) obtained from aglycone ions of steryl glucosides [SG-Glc+H]^+^ identified at level 1.

Within the SG standard mixture, sitosteryl glucoside, campesteryl glucoside, and stigmasteryl as well as cholesteryl glucoside were all of sufficient intensity to undergo MS/MS analysis (Figure 6). Their spectral similarity score when compared to their corresponding FS were above 0.99 (Table 5) indicating that a score higher than 0.99 is representative of identical spectral data. Δ^5^-avenasteryl glucoside was not fully baseline separated from its isomer Δ^7^-avenasteryl glucoside in the SG standard mixture, thus, spectral purity could not be ensured and another source for this SG was required (see level 2 identified SG below).

**Table 5:** Spectral similarity scores between ESI-MS/MS spectra of steryl glucosides (SG) and their corresponding free sterols (FS) and isomeric forms.

**SG**	**Corresponding FS**	**Isomeric forms**
Sitosteryl glucoside	1.00	0.95 (Δ^7^-stigmastenyl glucoside)*^c^*
Stigmasteryl glucoside	0.99	0.98 (Δ^5^-avenasteryl glucoside)
		0.97 (Spinasteryl glucoside)
		0.97 (Poriferasta-7,25-dienyl glucoside)
Campesteryl glucoside	0.99	0.91 (Δ^7^-campesteryl glucoside)*^c^*
Cholesteryl glucoside	0.99	–*^b^*
24-Methylenecholesteryl glucoside	0.99	0.97 (Brassicasteryl glucoside)
Brassicasteryl glucoside	1.00	0.97 (24-methylenecholesteryl glucoside)
Δ^5^-Avenasteryl glucoside	0.98	0.98 (Stigmasteryl glucoside)
		0.95 (Spinasteryl glucoside)
		0.98 (Poriferasta-7,25-dienyl glucoside)
Sitostanyl glucoside	0.97	–*^b^*
Spinasteryl glucoside	0.99	0.95 (Δ^5^-avenasteryl glucoside)
		0.97 (Stigmasteryl glucoside)
		0.95 (Poriferasta-7,25-dienyl glucoside)
Poriferasta-7,25-dienyl glucoside	–*^a^*	0.98 (Δ^5^-avenasteryl glucoside)
		0.97 (Stigmasteryl glucoside)
		0.95 (Spinasteryl glucoside)
Δ^7^-stigmastenyl glucoside*^c^*	–*^a^*	0.95 (Sitosteryl glucoside)
Δ^7^-campesteryl glucoside*^c^*	–*^a^*	0.91 (Campesteryl glucoside)

a *No FS standard available*.

b *No isomeric form analyzed*.

c *These SG were at very low concentration in the food source*.

As shown for their respective FS, the MS/MS spectra were characterized by dominant *m/z* 147 and *m/z* 161 indicating their Δ^5^-structure. The loss of side chain resulted in *m/z* 257 in case of sitosteryl glucoside and campesteryl glucoside and in *m/z* 255 for stigmasteryl glucoside indicating its C22-unsaturated side chain. Cholesteryl glucoside was characterized by *m/z* 259 being more abundant than fragment *m/z* 257, the latter of which being assigned to side chain loss as found for its FS. Stigmasteryl glucoside was characterized further by the dominant appearance of *m/z* 297, another trait distinguishing unsaturated side chain sterols from saturated side chain sterols.

#### Level 2 identified SG−24-methylenecholesteryl glucoside, brassicasteryl glucoside, Δ^5^-avenasteryl glucoside, sitostanyl glucoside, and spinasteryl glucoside

**Figure 7:**
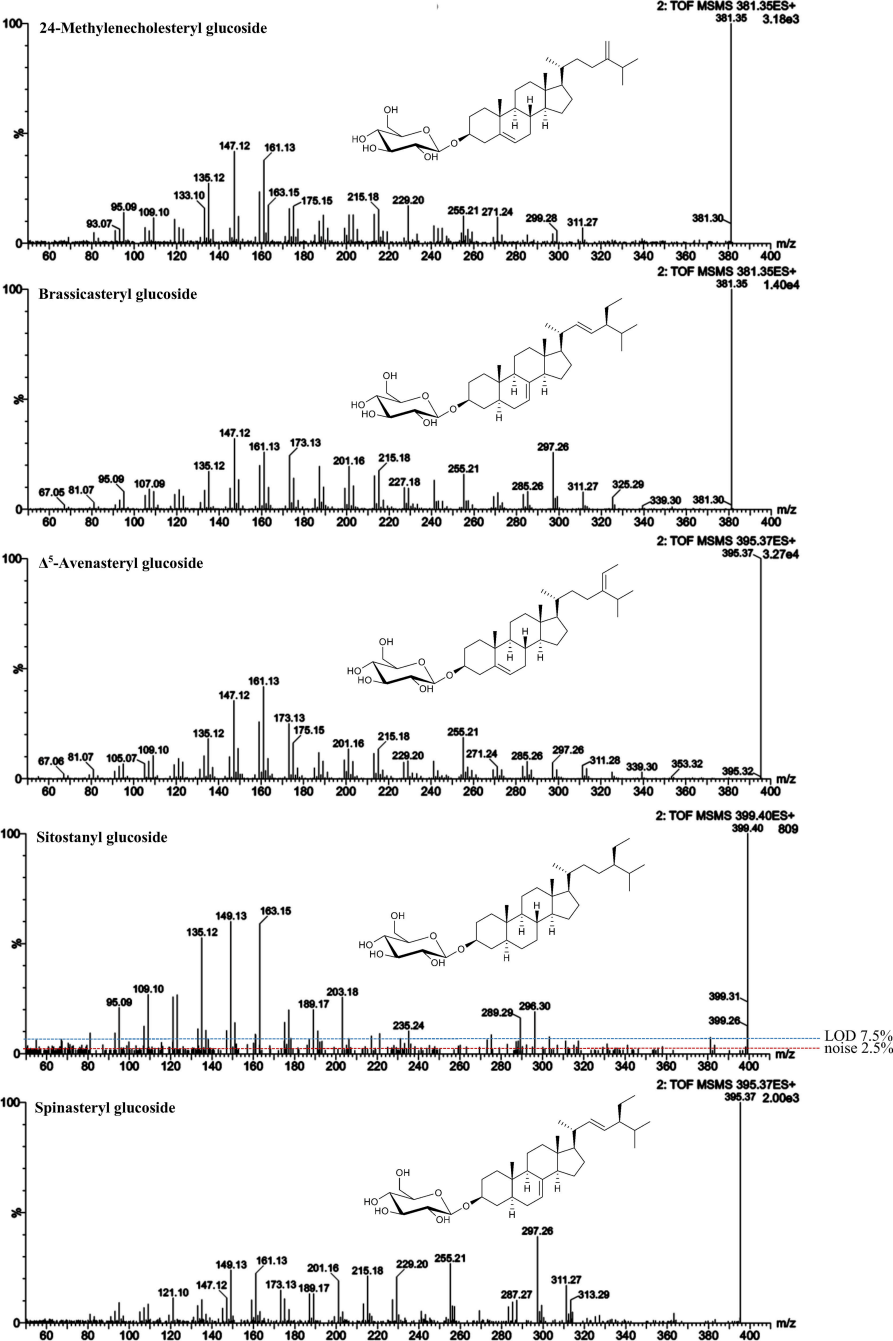
ESI-MS/MS spectra (25 V) obtained from aglycone ions of steryl glucosides [SG-Glc+H]^+^ extracted from different food sources identified at level 2. In case of sitostanyl glucoside, noise level and limit of detection (LOD) are indicated.

Based on above presented data from FS, five SG from different food sources were identified and their MS/MS spectra were obtained (Figure 7). In rapeseeds, two peaks with *m/z* 583 as sodiated SG adducts (*m/z* 381 as aglycone ions) were detected, of which one was expected to be brassicasterol glucoside. 24-Methylenecholesterol is an isomer of brassicasterol and eluted prior to brassicasterol based on the FS data presented above. RRT of those sterols as FS and RRT assessed as SG were identical and implied that the peak at RRT 0.97 could be assigned to 24-methylenecholesteryl glucoside and the peak at RRT 0.99 to brassicasteryl glucoside. For both, the spectral similarity score when compared to their corresponding FS was 0.99 (Table 5). Spectral similarity between the two SG was 0.97 (Table 5) indicating only minor differences between their spectra. Spectral data from brassicasteryl glucoside revealed the presence of a dominant *m/z* 297, which was much less pronounced in case of 24-methylenecholesteryl glucoside. Same observation was made for their respective FS (Figure 3). Both were however characterized by dominant *m/z* 147 and *m/z* 161 for C-ring fragmentation and *m/z* 255 for side chain loss confirming their Δ^5^-form in combination with an unsaturated side chain. 24-Methylenecholesterol as a glucoside derivative has only been found recently in microalgae by GC-MS analysis that omitted acid hydrolysis. Containing a methylidene side chain that is highly prone to isomerization under acidic conditions caused by the same underlying mechanism as found for ethylidene side chain sterols (Münger and Nyström, 2014; Münger et al., 2015) is most likely the reason why 24-methylenecholesterol as a glycosylated sterol has not been observed in higher plants before. 24-Methylenecholesterol as FS was reported to occur in different vegetable oils with soybean oil having the highest concentration followed by rapeseed oil, whereas it was absent in sunflower seed, peanut and safflower seed oil (Xu et al., 2018). Other food sources are rice and olives (Omoloye and Vidal, 2007; Ammar et al., 2017).

Δ^5^-Avenasteryl glucoside was highly abundant in oat bran being the main SG with *m/z* 597. Again, the predominance of *m/z* 255 over *m/z* 257 confirmed that Δ^24(24^1^)^ resulted in the same fragment ion as in the case of Δ^22^-unsaturation due to side chain loss. Its spectral similarity score to Δ^5^-avensterol as FS also extracted from oat was with 0.98 (Table 5) somewhat lower than when a pure FS standard was available.

Sitostanyl glucoside was detected as sodiated adduct (*m/z* 601) in wheat bran (Figure 4B) and its ESI-MS/MS (Figure 7) resembled the spectrum obtained from sitostanol as FS (Figure S14), with the RRT being very close as well (1.07 for FS and 1.08 for SG). Its distinctive spectral features were *m/z* 149 and 163, fragment ions that were reflective of stanols as FS. Also, *m/z* 135 was among the highest intensity fragments comparable to what was observed for sitostanol as FS. In this case, spectral similarity score when compared to the MS/MS spectrum of sitostanol as FS was lower than for the other level 2 identified SG (0.97). The reason may be attributed to its low concentration within the wheat bran sample resulting in a MS/MS spectra with fragment ions at intensities below the limit of detection (LOD; defined as 3 × noise level). This underlines the difficulty of identifying minor SG as they mostly appear at very low concentration in foods together with other SG that occur at higher levels. Simply increasing sample size will not solve this issue as this would result in overloading the LC-column and intensifying matrix effects.

Spinasteryl glucoside was confirmed in pumpkin seeds based on the analysis of spinasterol as FS by identical ESI-MS/MS spectra (spectral similarity score of 0.99, Table 5) with its dominant *m/z* 149, 161, 255, and 297 and by knowing that it elutes prior to its Δ^5^-form, stigmasteryl glucoside, as shown for their respective FS. Same as for its FS, *m/z* 149 confirmed its Δ^7^-structure while *m/z* 255 and 297 were reflective of its Δ^22^-double bond.

Spinasteryl glucoside (Figure 7), Δ^5^-avenasteryl glucoside (Figure 7) and stigmasteryl glucoside (Figure 6) are isomeric compounds and their spectral similarity score among each others ranged from 0.95 to 0.98 (Table 5). Δ^5^-Avenasteryl glucoside (RRT 1.00) and stigmasteryl glucoside (RRT 1.03) only differ in the position of the double bond in the side chain showed highly similar spectra (0.98), thus, their distinction is only possible in case of chromatographic separation as was the case for these two isomers (Figure 5, g and j).

#### Level 3 identified SG—Δ^7^-campesteryl glucoside, Δ^7^-stigmastenyl glucoside, poriferasta-7,25-dienyl glucoside, poriferasta-7,22,25-trienyl glucoside, Δ^7^-avenasteryl glucoside, cholestanyl glucoside and campestanyl glucoside

**Figure 8:**
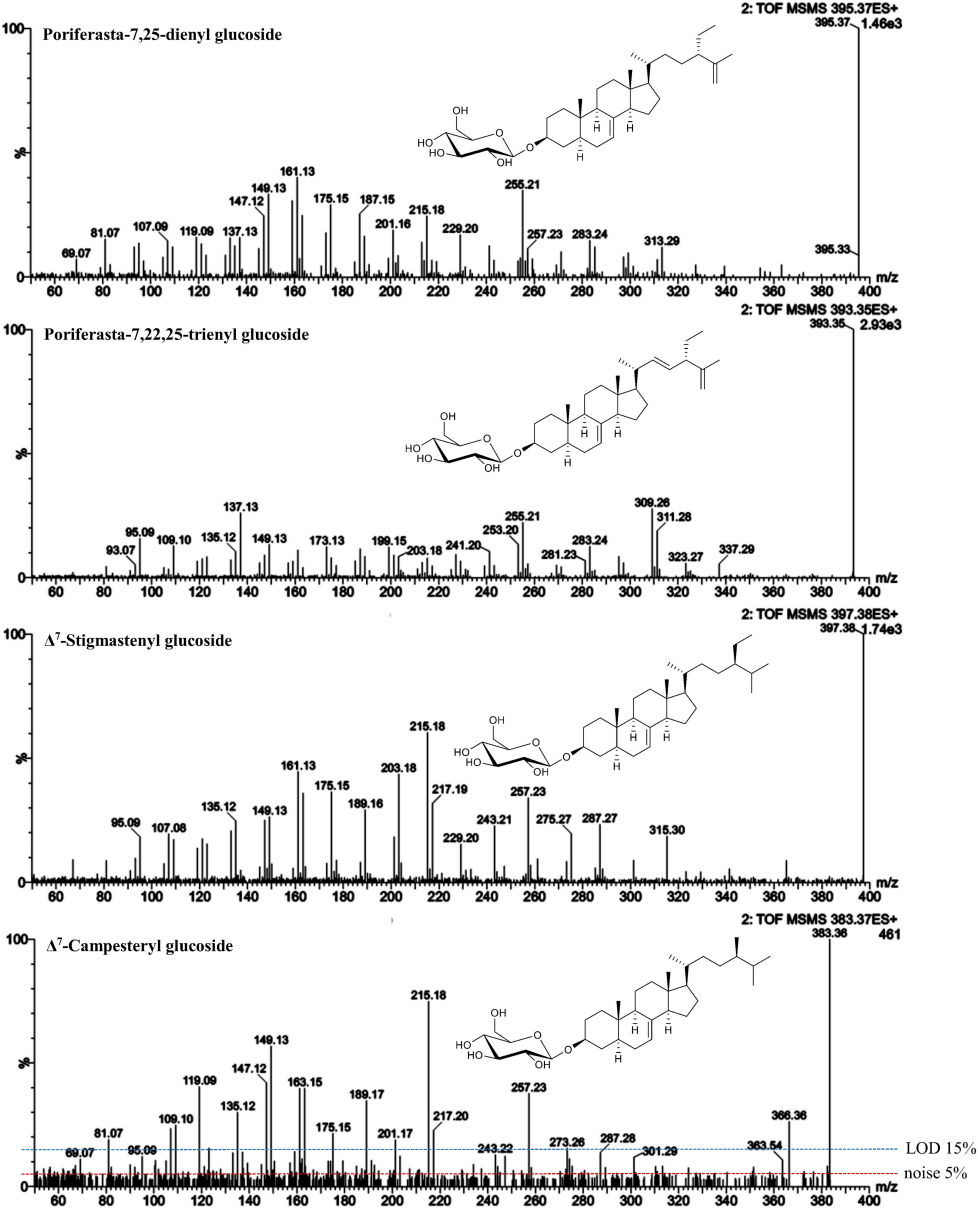
ESI-MS/MS spectra (25 V) obtained from aglycone ions of steryl glucosides [SG-Glc+H]^+^ extracted from different food sources identified at level 3. In case of Δ^7^-campesteryl glucoside, noise level and limit of detection (LOD) are indicated.

In case of Δ^7^-stigmasteryl glucoside and Δ^7^-campesteryl glucoside, no isomeric forms were detected as sodiated adducts in the melon sample, thus it represented a good source for the acquisition of their ESI-MS/MS spectra (Figure 8). The isomeric form of spinasteryl glucoside in pumpkin seed samples was identified as poriferasta-7,25-dienyl glucoside due to known elution order and previous knowledge about the occurrence in *Cucurbitaceae* (Breinhölder et al., 2002; Strobl, 2004; Münger et al., 2015). Poriferasta-7,22-25-trienyl glucoside occurs almost exclusively in *Cucurbitaceae* and only one single peak was found as sodiated adduct with *m/z* 595 in pumpkin seeds. Δ^7^-Steryl glucoside identified at level 3 were characterized by *m/z* 149 higher than *m/z* 147 derived from C-ring fragmentation (Figure 8), which was already shown to be a characteristic trait of spinasterol among the FS analyzed (Figure 3). Side chain loss resulted in *m/z 257* in case of Δ^7^-stigmastenol glucoside and Δ^7^-campesterol glucoside and *m/z 255* for poriferasta-7,25-dienyl and poriferasta-7,22,25-trienyl glucoside, two sterols with unsaturated side chain. For both of them, *m/z* 297 was not abundant most likely due to the position of the double bond within the side chain for poriferasta-7,25-dienyl glucoside and due to two double bonds in case of poriferasta-7,22,25-trienyl glucoside, inducing a different pathway for partial side chain fragmentation. Poriferasta-7,22,25-trienyl glucoside was further characterized by a strong *m/z* 309. Additionally, when comparing Δ^7^-stigmastenyl glucoside (Figure 8) to its Δ^5^-form, sitosteryl glucoside (Figure 5), high intensity of *m/z* 215 in case of the Δ^7^-sterol seemed to be an additional fragment ion that enables their differentiation.

Similarity score of Δ^7^-campesterol glucoside and Δ^7^-stigmasterol glucoside to their isomeric Δ^5^-forms were 0.91 and 0.95, respectively (Table 5). These low levels may however not be attributed to the differences in fragmentation patterns but rather due to the low concentration of the Δ^7^-sterols in the food samples, which resulted in a low intensity precursor ion with fragment ions below LOD as shown for Δ^7^-campesteryl glucoside (Figure 8). From this we can conclude that similarity scores may only serve as a valuable tool for SG that occur at reasonable concentration (precursor ions must reach an intensity of at least 10^4^ in MS mode). Minor SG always occur along with major SG at high concentrations, thus, in order to avoid overloading the LC-column, purification steps to obtain single SG species would be of prime importance i.e., using preparative LC.

Δ^7^-Avenasteryl glucoside was detected based on the *m/z* of the sodiated ion in melon, pumpkin seeds and the SG standard, however, the intensity of the aglycone ion was not sufficient (below 10^4^) to generate a reliable ESI-MS/MS spectrum. The same was the case for cholestanyl glucoside (detected in potato peel) and campestanyl glucoside (detected in SG standard mixture and wheat bran).

#### Level 4 identified SG—citrostadienyl glucoside

**Figure 9:**
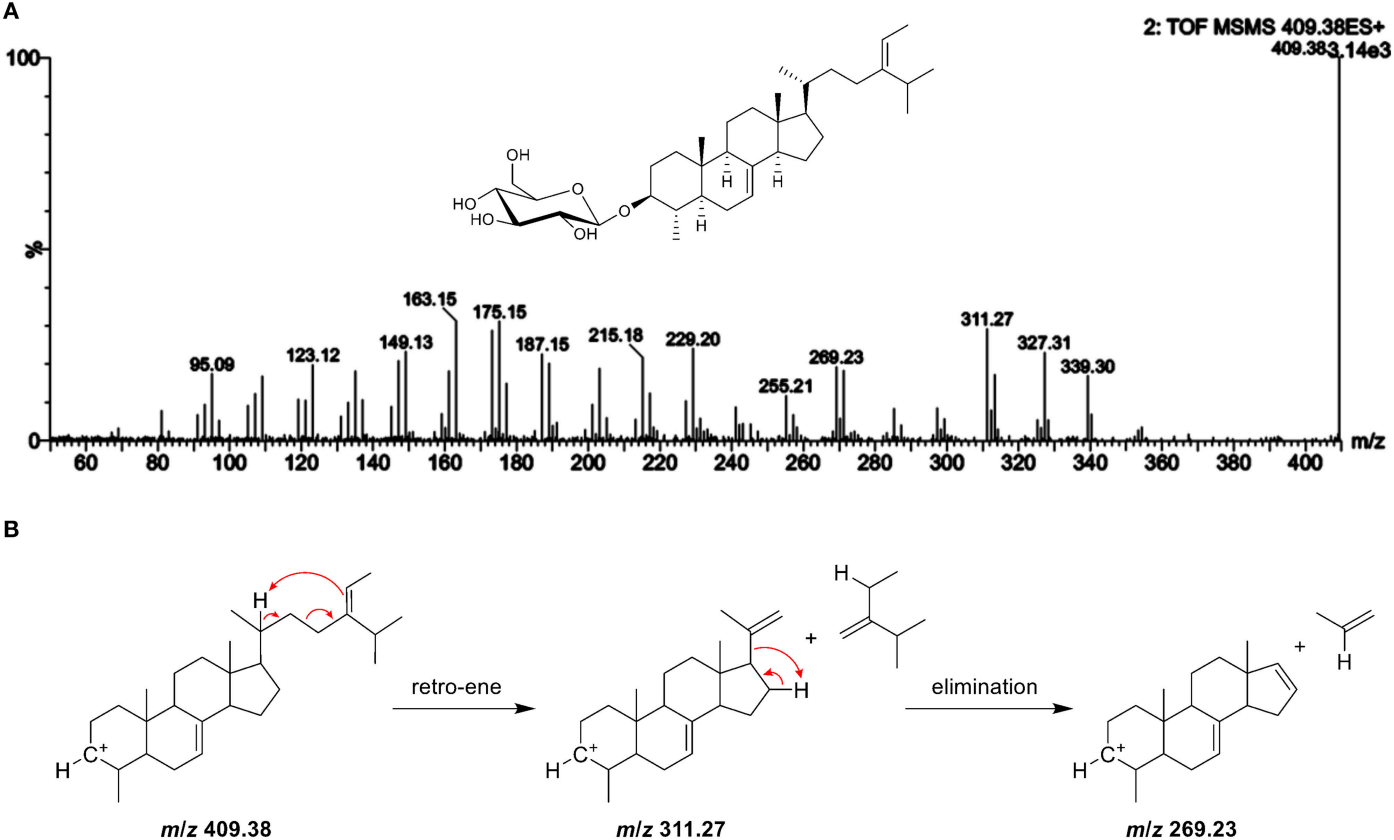
**(A)** ESI-MS/MS spectra of the aglycone ion *m/z* 409 at 25 V of a novel steryl glucoside (SG) found in potato peel with *m/z* 611 as sodiated adduct suggesting to be a 4-monomethyl sterol (level 4 identification); additional GC-MS experiments revealed that the compound can be attributed to citrostadienyl glucoside (see Figure S16); **(B)** the proposed fragmentation pathway for the formation of the characteristic fragment ion with *m/z* 269.

In potato peel, the ion with *m/z* 611 was at high intensity and eluted in the SG region (Figure 5D, peak m). SG with masses higher than *m/z* 601 are assumed to be either 4-monomethyl or 4,4-dimethylsterols, however, these have not yet been found to occur as SG in higher plants and literature suggests that 4-desmethyl sterols are exclusive substrates for the biosynthesis (Wojciechowski, 1991; Potocka and Zirnowski, 2008). Opposed to these earlier findings about SG, the compound with *m/z* 611 was indeed a glycosylated sterol as it formed an aglycone ion due to the loss of glucose. The ESI-MS/MS spectra resembled other sterol spectra and it eluted in the region of other SG. Mass accuracy was 0.2 ppm and thus confirmed elemental composition of C_36_H_60_O_6_Na, being the sodiated adduct of a glycosylated C30-sterol. In the FS analyses, only one dimethylsterol was included (lanosterol, Figure S7), which is characterized by identical elemental composition as the SG found in potato peel. However, the RRT of lanosterol (RRT 1.01) and of *m/z* 611 in potato peel (RRT 1.04) differed as did their spectral data (similarity score was 0.92). Characteristic fragment ion of the aglycone in the MS/MS spectra of this novel SG was *m/z* 269 (Figure 9), which corresponds to the ion formed by the loss of the side chain within a monomethyl sterol. Not being able to confirm the identify of *m/z* 611 by ESI-MS/MS using a FS standard, the potato peel SG fraction was subjected to enzymatic hydrolysis and further analyzed by GC-MS, for which spectral data are more complete (Figure S17). Indeed, a 4-monomethyl sterol was observed and was identified as citrostadienol based on database search (Kamal-Eldin et al., 1992).

Citrostadienol is a Δ^7^-sterol with an ethylidene side chain, which explains why this SG has not been detected before. In general, Δ^7^-sterols and alkene side chain sterols undergo isomerization during acid hydrolysis that is required for the analysis of SG as FS by GC (Münger et al., 2015). Based on the labile structure, acid hydrolysis will result in artifact production of citrostadienyl glucoside and the compound will, therefore, not be assessed. Citrostadienol occurs as FS in several foods such as olive oil (Itoh et al., 1973; Damirchi et al., 2005; Lukic et al., 2015), pomegranate seeds (Verardo et al., 2014), bilberry *Vaccinium myrtillus* (Szakiel et al., 2012), and sea buckthorn (Li et al., 2007). Among different vegetable oils, it was highest in sunflower seed oil (Xu et al., 2018). Further investigations should be carried out in order to identify them as sources for citrostadienyl glucoside. Recently, a 4-monomethyl steryl glucoside was found by direct GC-MS in the microalga *K. veneficum* based on the ion with *m/z* 269 that indicated a methyl group at C4 (Yu et al., 2017), corroborating the observations made within this study.

## Conclusion

The unavailability of SG standard compounds remains a limitation in the unequivocal identification of these sterol conjugates. Nonetheless, not only major but also minor SG species deserve to be included in SG profiling studies as a wide range of SG occur in our diet. In comparison to SG analysis with GC-MS in EI mode, ESI provides softer ionization and allows the direct analysis of the polar sterol conjugates without being hydrolysed and derivatized prior to analysis, which is useful in straightforward profiling studies. We have presented a novel approach to identify SG at different levels of identification that also included the analysis of FS, for which identical spectra are generated in ESI-MS/MS. The prior investigation on fragmentation patterns obtained by ESI-MS/MS of FS enabled to identify fragment ions that characterized structural features within the sterol moiety despite high structural similarity. Elution order and spectral data from FS were directly transferable to their related SG when analyzed as aglycone ions. At the same time, the glycosidic nature of SG could be confirmed by their analysis as sodiated adducts. Using UPLC-ESI-MS/MS, two novel SG were detected in higher plants that were not found with traditional methods due to their acid-labile structure. In addition, these findings suggest for the first time that 4-monomethyl sterols also occur as glycosylated conjugates in higher plants. Overall, spectral ESI-MS/MS data presented here may serve as fundamental basis for a spectral library for SG profiling.

## Author contributions

LM and LN conceived the idea and designed the work. LM performed all experiments. LM and SB worked on data analysis and interpretation. LM and SB wrote the paper. All authors contributed to the manuscript and approved the final version.

### Conflict of interest statement

The authors declare that the research was conducted in the absence of any commercial or financial relationships that could be construed as a potential conflict of interest.

## Abbreviations

APCI: atmospheric pressure chemical ionization
ASG: acylated steryl glucosides
EIC: extracted ion chromatogram
ESI: electrospray ionization
FS: free sterols
GC: gas chromatography
LOD: limit of detection
MS: mass spectrometry
*m/z*: mass-to-charge-ratio
RRT: relative retention time
SE: steryl esters
SG: steryl glucosides.
