# Supplementary material for: UPLC-MS/MS Based Identification of Dietary Steryl Glucosides by Investigation of Corresponding Free Sterols
Source: Front Chem. 2018 Aug 22;6:342. doi: 10.3389/fchem.2018.00342 (PMC6113793; doi:10.3389/fchem.2018.00342)
Supplement: Supplementary file 1 [file Data_Sheet_1.pdf]

Supplementary Material

UPLC-MS/MS based identification of dietary steryl glucosides by investigation of corresponding free sterols

Linda H. Münger[1], Samy Boulos[1], Laura Nyström[1*]

[1] Laboratory of Food Biochemistry, Institute of Food, Nutrition and Health, Department of Health Sciences and Technology, ETH Zurich, Zurich, Switzerland

[*] **Correspondence:**

Prof. Dr. Laura Nyström
laura.nystroem@hest.ethz.ch

Contents:

1) **Spectral similarity score**……………………………………………………………1-2
2) **ESI-MS/MS spectra of FS**…………………………………………………………..3-8
3) **Fragmentation pathways**…………………………………………………………...9
4) **GC-MS analysis of SG from potato peel after enzymatic hydrolysis**…………10

1) Spectral similarity score

Table S1 Selection of relevant mass-to-charge-ratios (*m/z*) used for spectral similarity score calculations.

[*m/z*]
81.071
95.086
109.102
121.102
123.118
131.087
133.102
135.118
145.102
147.118
149.134
159.118
161.133
163.147
173.133
175.149
177.162
185.133
187.148
189.165
191.181
199.149
201.164
203.18
205.196
213.165
215.18
217.196
241.196
243.212
253.196
255.212
257.228
259.243
295.243
297.259
309.258
311.274
313.289
Precursor ion

Table S2 Spectral similarity scores between non-isomeric and isomeric FS ordered by decreasing masses; scores higher than 0.99 indicating equal spectra are highlighted in bold; isomeric sterols are labelled with identical letters; for calculation of similarity score among all FS the precursor ion was not included.

	Lanosterol	Sitostanol	Sitosterol	Δ^5-Avenasterol[a]	Spinasterol[a]	Stigmasterol[a]	Campesterol	Brassicasterol[b]	24-Methylenecholesterol[b]	Ergosterol	Coprostanol[c]	Cholestanol[c]	Cholesterol	7-Dehydrocholesterol[d]	Desmosterol[d]
Lanosterol	-	0.83	0.88	0.84	0.84	0.85	0.87	0.86	0.90	0.70	0.84	0.86	0.86	0.72	0.90
Sitostanol	0.83	-	0.79	0.65	0.63	0.63	0.79	0.64	0.74	0.53	**0.99**	**0.99**	0.79	0.52	0.74
Sitosterol	0.88	0.79	-	0.89	0.81	0.85	0.99	0.86	0.93	0.71	0.81	0.81	0.98	0.72	0.95
Δ^5-Avenasterol[a]	0.84	0.65	0.89	-	0.94	0.97	0.89	0.97	0.98	0.83	0.68	0.69	0.88	0.89	0.96
Spinasterol[a]	0.84	0.63	0.81	0.94	-	0.98	0.82	0.97	0.93	0.80	0.67	0.68	0.80	0.84	0.90
Stigmasterol[a]	0.85	0.63	0.85	0.97	0.98	-	0.86	**0.99**	0.97	0.85	0.67	0.69	0.84	0.89	0.95
Campesterol	0.87	0.79	**0.99**	0.89	0.82	0.86	-	0.86	0.86	0.72	0.81	0.81	0.98	0.73	0.95
Brassicasterol[b]	0.86	0.64	0.86	0.97	0.97	**0.99**	0.86	-	0.97	0.86	0.68	0.70	0.85	0.90	0.96
24-Methylenecholesterol[b]	0.90	0.74	0.93	0.98	0.93	0.97	0.86	0.97	-	0.84	0.77	0.78	0.92	0.89	**0.99**
Ergosterol	0.70	0.53	0.71	0.83	0.80	0.85	0.72	0.86	0.84	-	0.57	0.60	0.71	0.91	0.84
Coprostanol[c]	0.84	**0.99**	0.81	0.68	0.67	0.67	0.81	0.68	0.77	0.57	-	**1.00**	0.81	0.55	0.76
Cholestanol[c]	0.86	**0.99**	0.81	0.69	0.68	0.69	0.81	0.70	0.78	0.60	**1.00**	-	0.81	0.58	0.78
Cholesterol	0.86	0.79	0.98	0.88	0.80	0.84	0.98	0.85	0.92	0.71	0.81	0.81	-	0.72	0.93
7-Dehydrocholesterol[d]	0.72	0.52	0.72	0.89	0.84	0.89	0.73	0.90	0.89	0.91	0.55	0.58	0.72	-	0.88
Desmosterol[d]	0.90	0.74	0.95	0.96	0.90	0.95	0.95	0.96	0.99	0.84	0.76	0.78	0.93	0.88	-

[a]sterols with *m/z* 395
[b]sterols with *m/z* 383
[c]sterols with *m/z* 371
[d]sterols with *m/z* 367

2) ESI-MS/MS spectra of free sterols (FS)

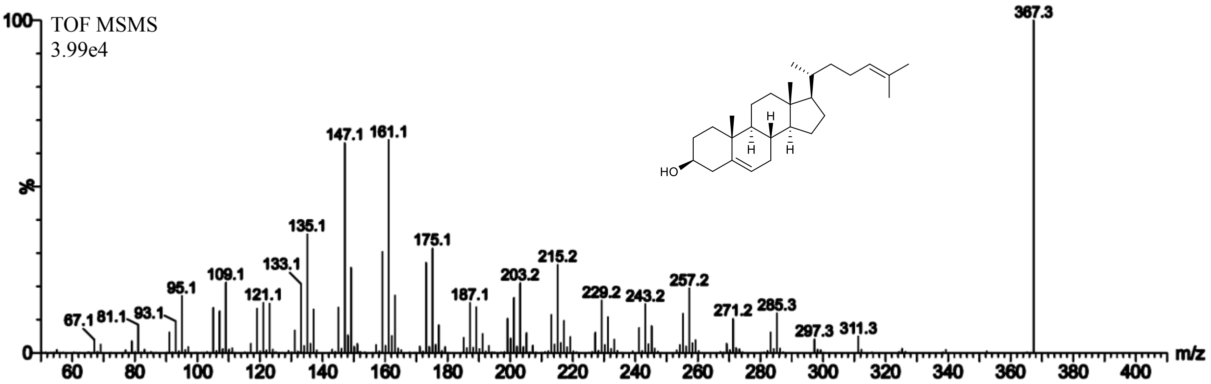

Fig. S1 ESI-MS/MS spectra of desmosterol at 25 V as $[FS\text{-}H_2O+H]^+$.

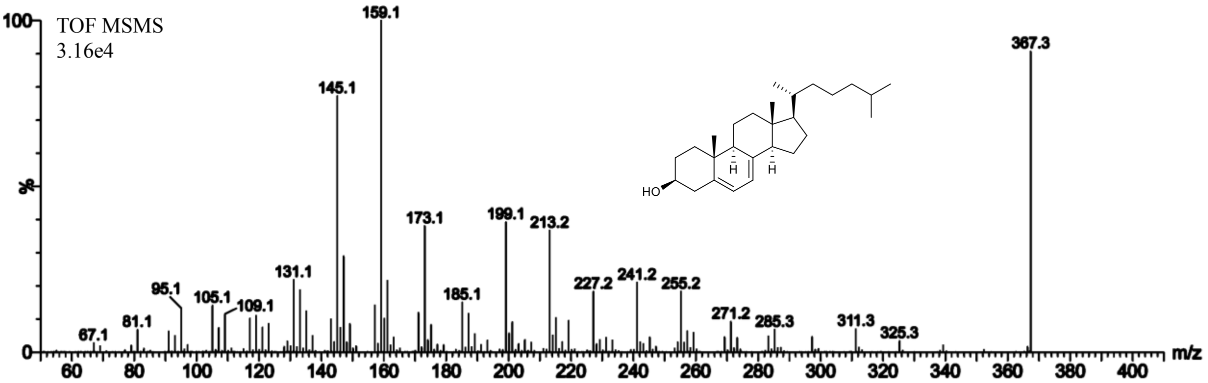

Fig. S2 ESI-MS/MS spectra of 7-dehydrosterol at 25 V as $[FS\text{-}H_2O+H]^+$.

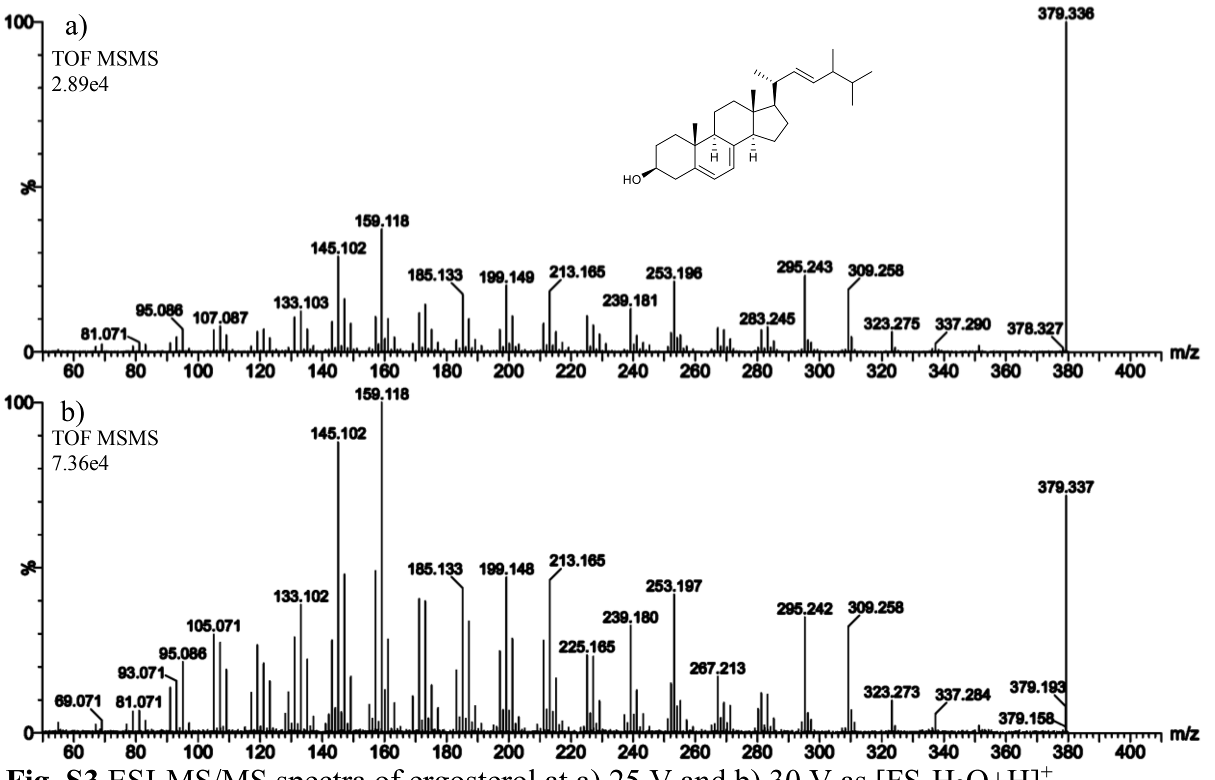

Fig. S3 ESI-MS/MS spectra of ergosterol at a) 25 V and b) 30 V as $[FS\text{-}H_2O+H]^+$.

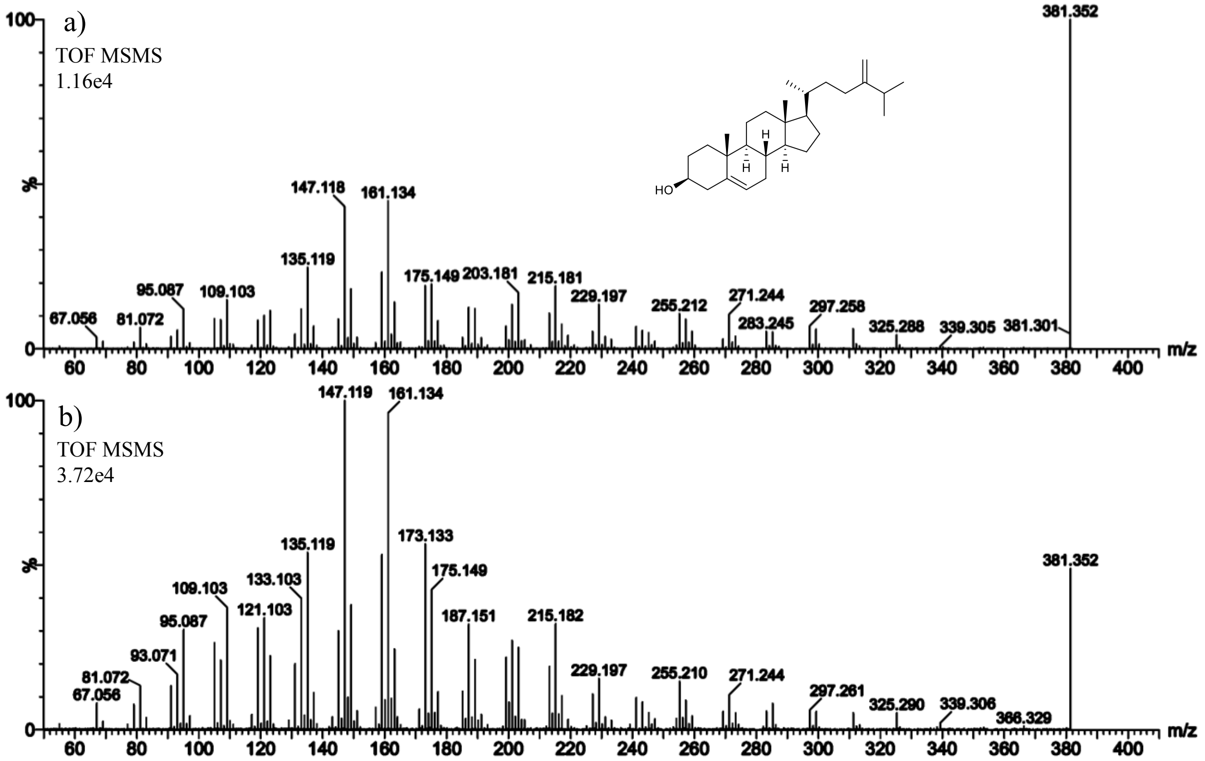

Fig. S4 ESI-MS/MS spectra of 24-methylenecholesterol at a) 25 V and b) 30 V as [FS-H_2O+H]$^+$.

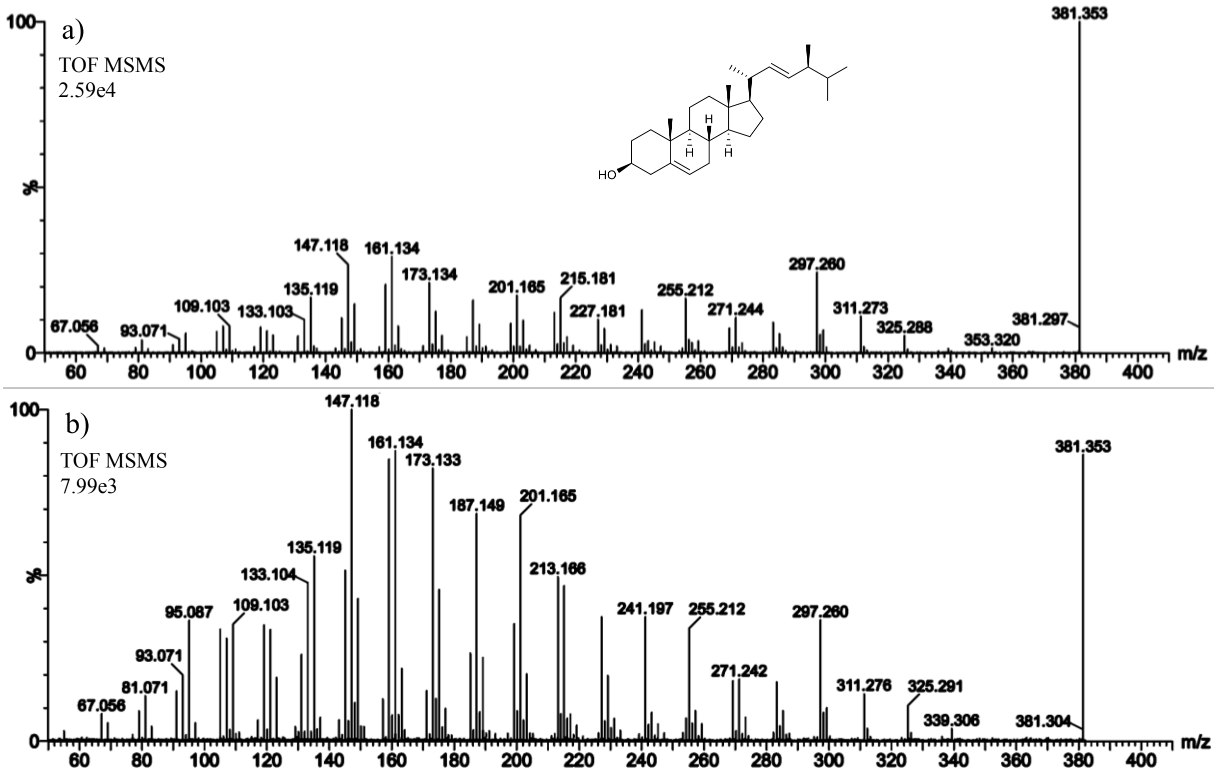

Fig. S5 ESI-MS/MS spectra of brassicasterol at a) 25 V and b) 30 V as [FS-H_2O+H]$^+$.

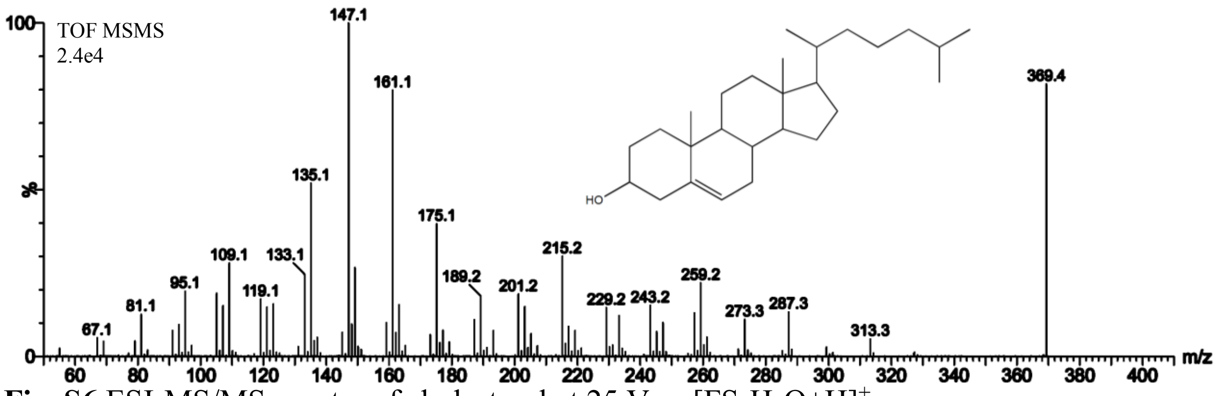

Fig. S6 ESI-MS/MS spectra of cholesterol at 25 V as $[FS\text{-}H_2O+H]^+$.

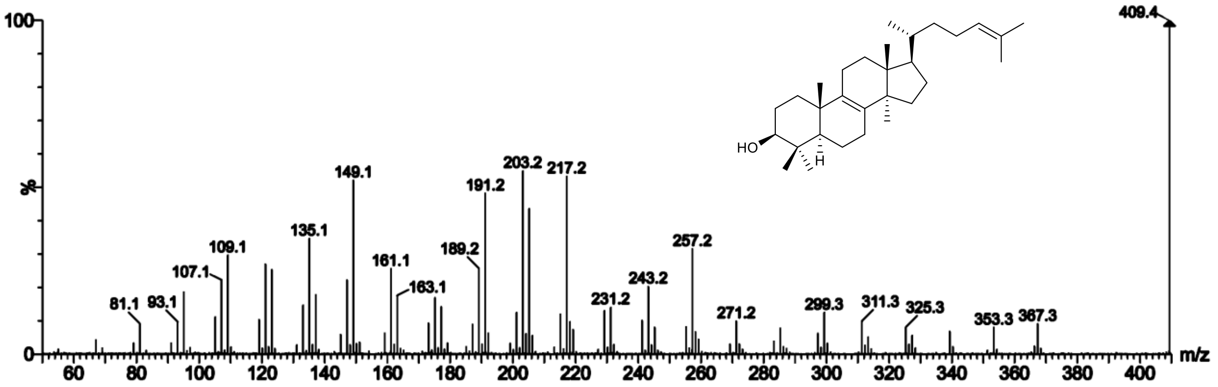

Fig. S7 ESI-MS/MS spectra of lanosterol at 25 V as $[FS\text{-}H_2O+H]^+$.

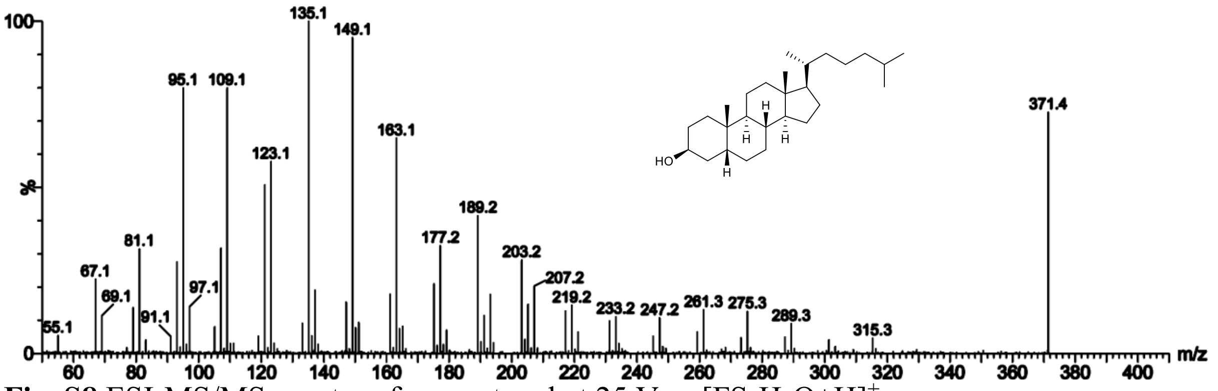

Fig. S8 ESI-MS/MS spectra of coprostanol at 25 V as $[FS\text{-}H_2O+H]^+$.

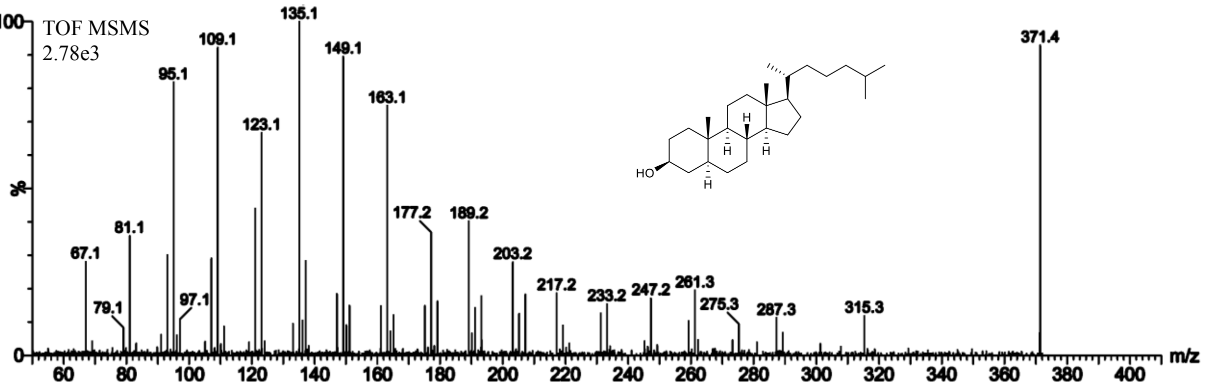

Fig. S9 ESI-MS/MS spectra of cholestanol at 25 V as $[FS-H_2O+H]^+$.

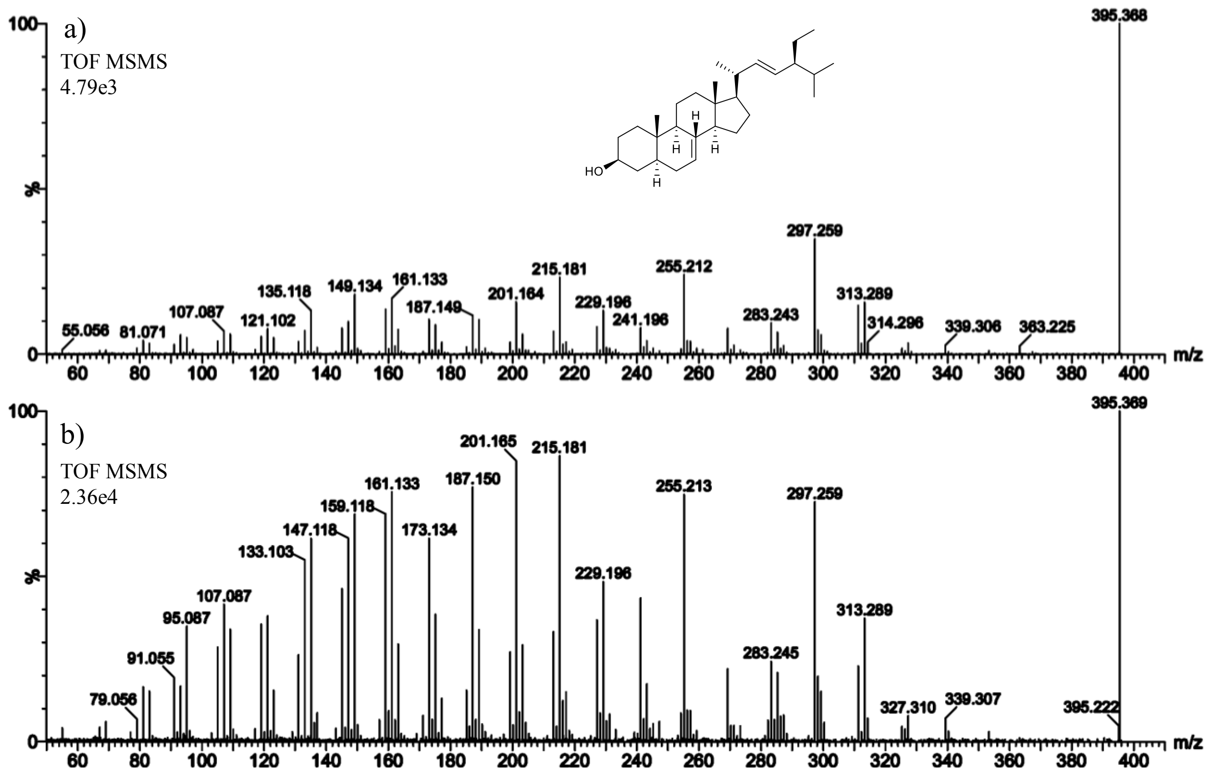

Fig. S10 ESI-MS/MS spectra of spinasterol at a) 25 V and b) 30 V as $[FS-H_2O+H]^+$.

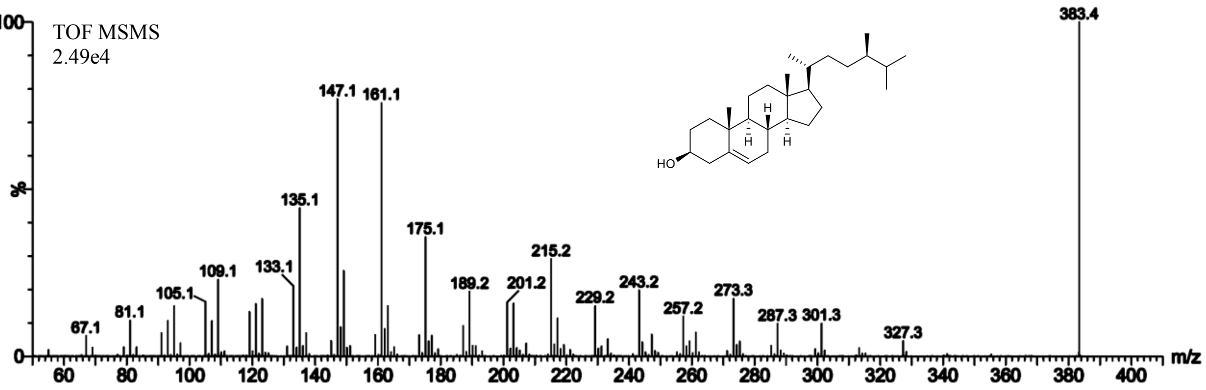

Fig. S11 ESI-MS/MS spectra of campesterol at 25 V as $[FS-H_2O+H]^+$.

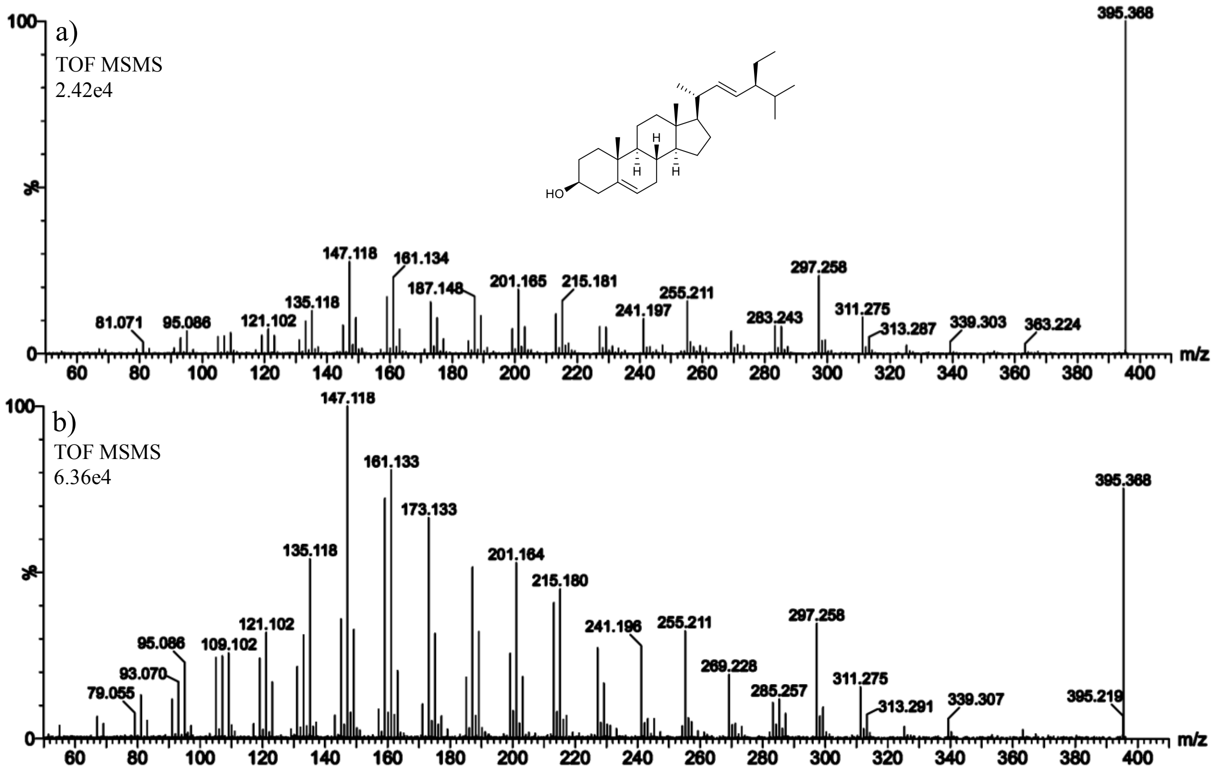

Fig. S12 ESI-MS/MS spectra of stigmasterol at a) 25 V and b) 30 V as $[FS\text{-}H_2O+H]^+$.

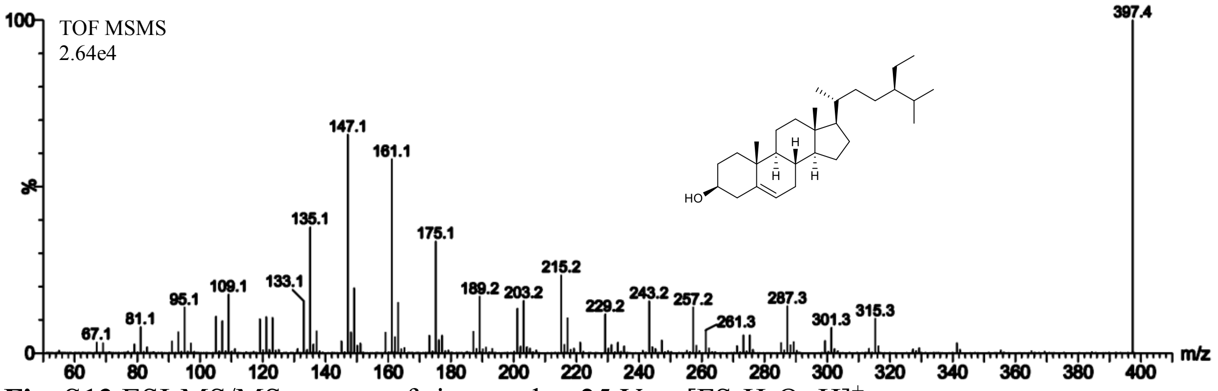

Fig. S13 ESI-MS/MS spectra of sitosterol at 25 V as $[FS\text{-}H_2O+H]^+$.

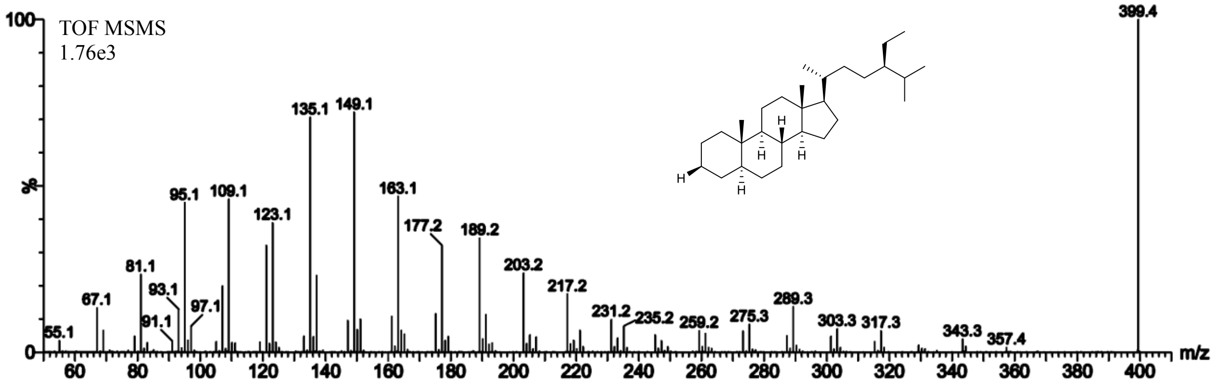

Fig. S14 ESI-MS/MS spectra of sitostanol at 25 V as $[FS\text{-}H_2O+H]^+$.

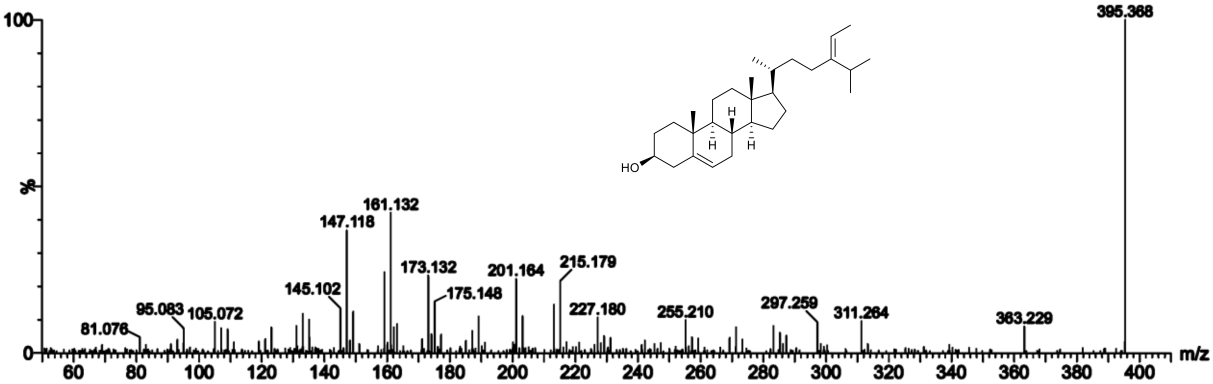

Fig. S15 ESI-MS/MS spectra of Δ^5-avenasterol from oat bran at 25 V as $[FS\text{-}H_2O+H]^+$.

3) Fragmentation pathways

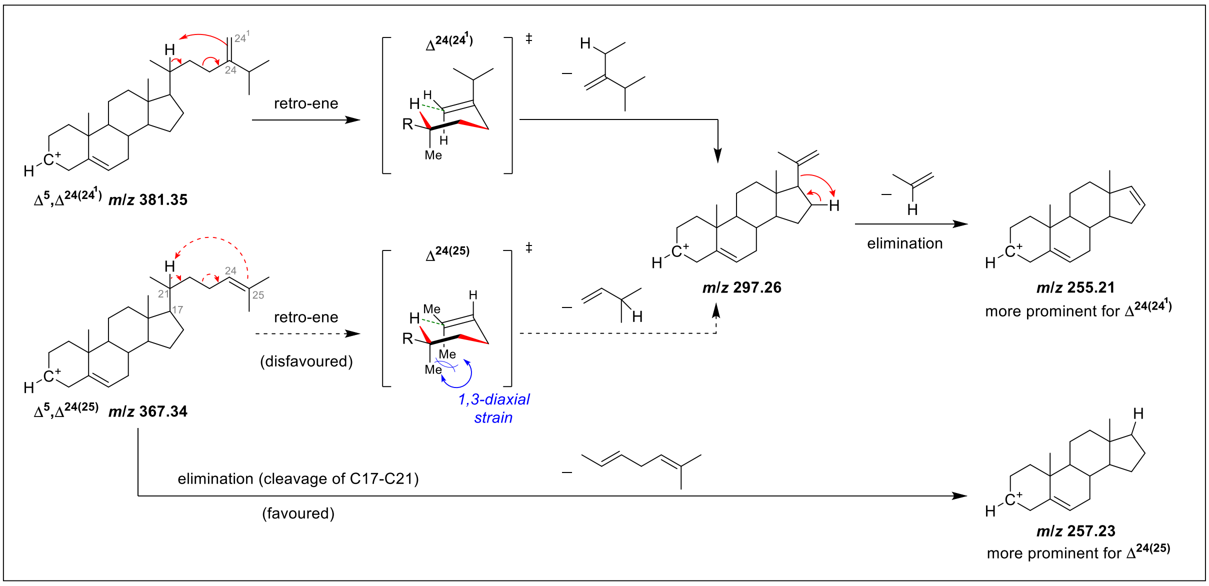

Fig. S16: The different preferred side chain fragmentation pathways depending on double bond position at C24(24^1) or C24(25) are shown on the examples of 24-methylenecholesterol (Δ^5,$\Delta^{24(24\ )}$; top left) and Δ^5-desmethylsterols (Δ^5,$\Delta^{24(25)}$; bottom left), respectively. The side chain fragmentation is induced by a retro-ene reaction, whereas the higher substituted C24(25) double bond leads to higher 1,3-diaxial strain of two methyl groups (Me↔Me) in the chair conformation-like transition state (‡), making this path less favorable compared to the sterol with the less substituted C24(24^1) double bond (Me↔H). The resulting partial side chain loss species *m*/*z* 297 is properly set up for an elimination reaction to the *m*/*z* 255 species, as observed for Δ^5,$\Delta^{24(24\ )}$-sterols. For Δ^5,$\Delta^{24(25)}$-sterols, however, the side chain loss preferentially follows the pathway analogous to sterols with saturated side chain (see Fig. 4G), leading to *m*/*z* 257 (bottom pathway). The same rationalization holds true for $\Delta^{24(24\ )}$- and $\Delta^{24(25)}$-sterols and their conjugates with additional C24^1-methyl or C24-methyl/ethyl groups.

4) GC-MS analysis of SG from potato peel after enzymatic hydrolysis

a)

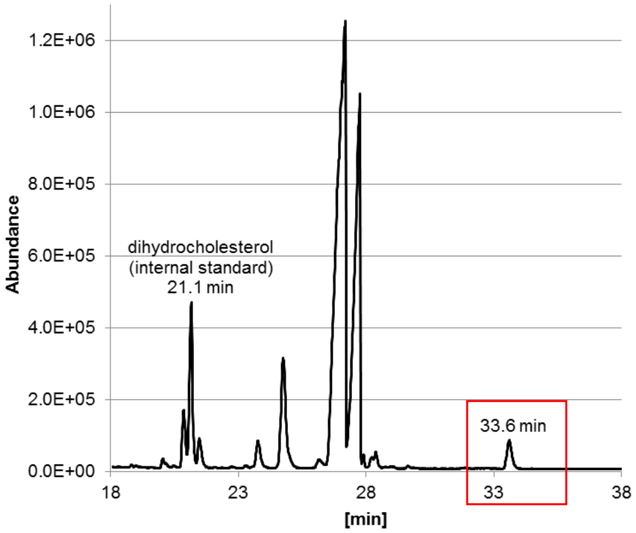

b)

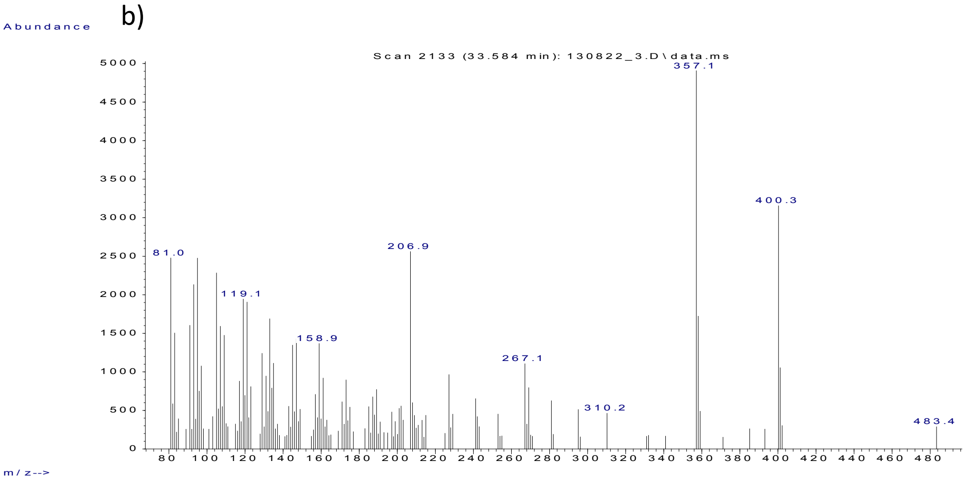

Fig. S17 a) GC chromatogram of free sterols (FS) as TMS derivatives obtained after enzymatic hydrolysis of steryl glucosides (SG) extracted from potato peel (based on procedure as in Münger and Nyström, 2014 [a]), sterol eluting at 33.6 min was identified as citrostadienol based on b) GC-MS spectrum of peak with retention time of 33.6 min; identified by comparison to published spectral data.[b]

[a]Münger, L.H., and Nyström, L. (2014). Enzymatic Hydrolysis of Steryl Glycosides for their Analysis in Foods. *Food Chem* 163**,** 202-211

[b]Kamal-Eldin, A., Appleqvist, L.A., Yousif, G., and Iskander, G.M. (1992). Seed Lipids of Sesamum-Indicum and Related Wild-Species in Sudan - the Sterols .3. *J Sci Food Agric* 59**,** 327-334
